# Sustainable use of low-cost adsorbents prepared from waste fruit peels for the removal of selected reactive and basic dyes found in wastewaters

**DOI:** 10.1007/s11356-024-31868-3

**Published:** 2024-01-27

**Authors:** Athanasia K. Tolkou, Eleftheria K. Tsoutsa, George Z. Kyzas, Ioannis A. Katsoyiannis

**Affiliations:** 1 Department of Chemistry, International Hellenic University, 65404 Kavala, Greece; 2 Department of Chemistry, Aristotle University of Thessaloniki, 54124 Thessaloniki, Greece

**Keywords:** Dye removal, Adsorption, Wastewater treatment, Peels, Natural adsorbents

## Abstract

Agricultural wastes are potential sustainable adsorbents since they are available in large quantities, are low-cost, and may require little or no treatment, in some cases. In this study, several fruit peels, such as banana, orange, and pomegranate, were collected from local markets and prepared by a simple and eco-friendly method and used as natural adsorbents for the removal of both anionic (Reactive Red 120 (RR120), Reactive Black 5 (RB5), Remazol Brilliant Blue R (RBBR)) and cationic Methylene Blue (MB) dyes found in wastewaters. Many industries, such as leather and textiles, can release huge amounts of synthetic dyes into the wastewater during dyeing processes. These are one of the most important pollutants of water pollution as they cause enormous damage to the water body and also affect the health of organisms due to their toxicity and carcinogenicity. The search for a sustainable and at the same time efficient material for the removal of a wide variety of dyes is the innovation of this work. These peels were prepared by washing, drying, grinding, and finally sieving, under natural sustainable conditions. Porosometry (BET analysis), FTIR, SEM/EDS, and XRD techniques were used to characterize the fruit peels before and after the adsorption process. Factors affecting the adsorption of dyes (adsorbent dosage, pH solution, initial concentration of dyes, contact time, and temperature) were investigated. According to the results, in terms of the effectiveness of fruit peels as (natural) adsorbent materials, for anionic dyes, 5.0–6.0 g/L of banana or orange dry peels was sufficient to remove near or even more than 90% anionic dyes at pH 2.0, and 4.0 g/L was sufficient to remove 98% of cationic MB dye at pH 9.0. Similar amount of pomegranate peels had lower efficiency for anionic dyes (50–70%), while cationic MB was still efficiently removed (98%) at pH 9.0. Moreover, the adsorption process in all cases was found to better fit to pseudo-second-order model, in comparison to pseudo-first-order model. According to isotherms, Freundlich model fitted better in some cases to the equilibrium data, while the Langmuir model in others. Finally, this study demonstrates the viability of reusing the banana, orange, and pomegranate peel adsorbents for eight, four, and five cycles, showing a gradual reduction of around 50% of their effectiveness.

**Graphical Abstract:**

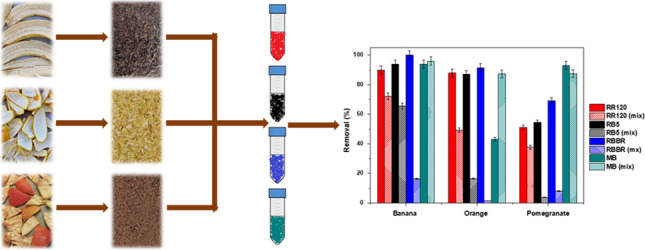

## Introduction

The access to clean water is vital for the well-being of humanity. Unfortunately, in many developing countries, over 1 billion of people is denied access to drinking water (Thillainayagam et al. 2022; Zhao et al. 2022). The pollution of industrial wastewater effluents, such as leather and textiles, represents a significant environmental challenge (Tolkou and Zouboulis 2020), particularly due to the presence of highly hazardous substances, such as synthetic dyes, which have the potential to cause carcinogenic effects (Mahmoudabadi et al. 2019), containing some dangerous constitutes, i.e., benzidine, 4-aminobiphenyl, and 4-aminoazobenzene, found in food colorant substances (Ghalkhani et al. 2022). This problem is more evident in countries that still dominate dye industry such as China, Malaysia, Bangladesh, Thailand, and Indonesia (Islam and Dey 2015; Li et al. 2023). This type of pollution mainly originates from industrial wastewater that contains a substantial concentration of pollutants. There are three types of dyes: non-ionic, cationic, and anionic. All of them are soluble in water and can pose harm even in small amounts (Panda et al. 2021). The dyes can be classified as natural or synthetic, with synthetic dyes being used more often and being available in a wide range of colors. Synthetic dyes are further categorized based on their specific applications, such as reactive, direct, disperse, basic, and vat dyes. They can also be classified based on their chemical structure and different functional groups, such as azo, anthraquinone, sulfur, oxazine, phthalocyanine, and triarylmethane dyes (Fobiri 2022; Yeow et al. 2021).

**Fig. 1:**
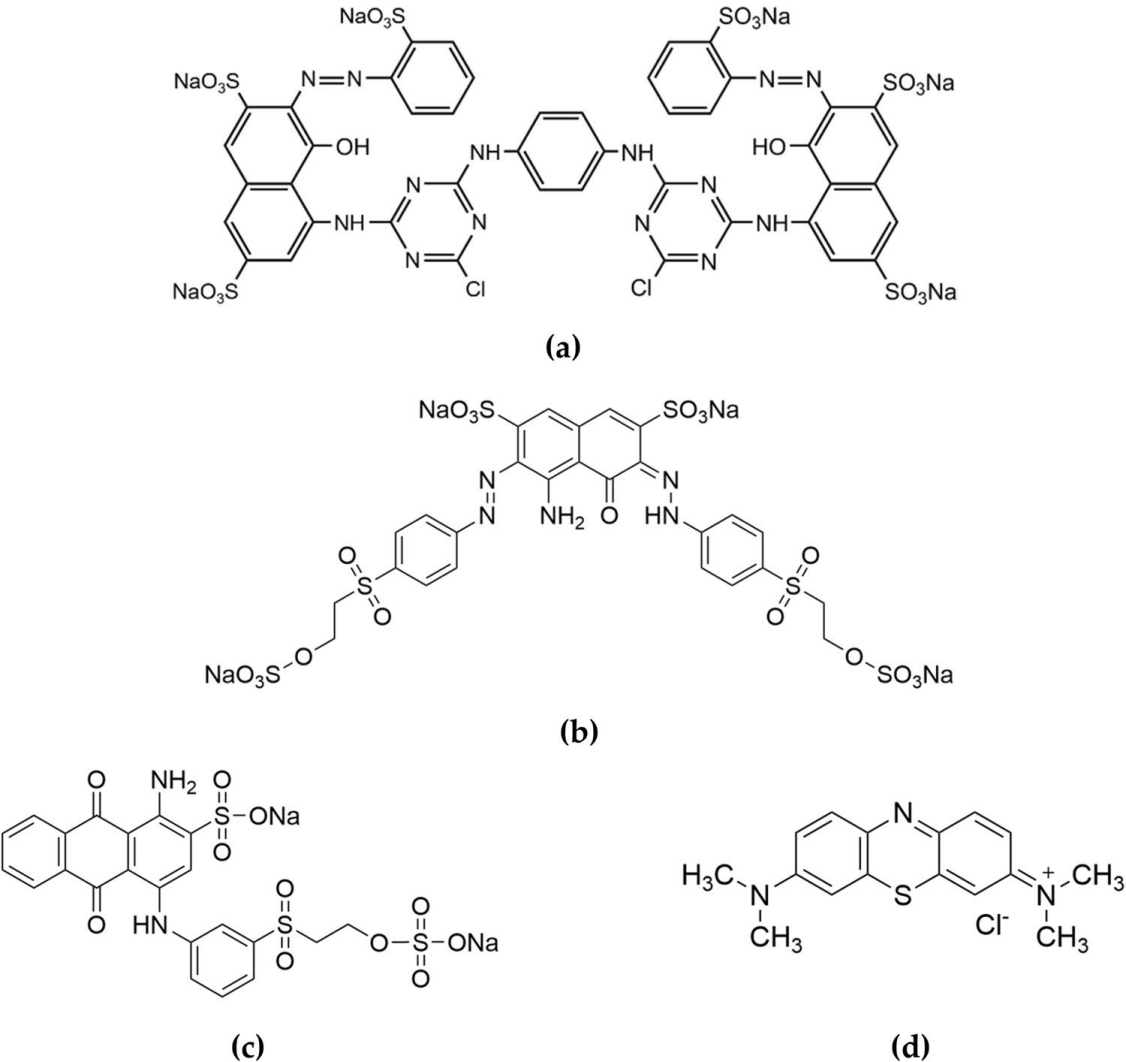
Structure formula of **a** Reactive Red 120 (RR120) (C_44_H_24_C_l2_N_14_O_20_S_6_Na_6_), **b** Reactive Black 5 (RB5) (C_26_H_21_N_5_Na_4_O_19_S_6_), **c** Remazol Brilliant Blue R (RBBR) (C_22_H_16_N_2_Na_2_O_11_S_3_), and **d** Methylene Blue (MB) (C_16_H_18_ClN_3_S)

Reactive Red 120, denoted hereafter as RR120 (C_44_H_24_C_l2_N_14_O_20_S_6_Na_6_) (Fig. 1a) and Reactive Black 5, denoted hereafter as RB5 (C_26_H_21_N_5_Na_4_O_19_S_6_) (Fig. 1c), are anionic azo dyes, being widely used in the textile industry to color cellulose-based fibers like cotton and rayon. These dyes have the unique property of forming a strong chemical bond with the fibers, creating a covalent linkage. However, the presence of synthetic azo dyes, including reactive dyes, in industrial processes like dyeing and textile production poses a significant risk to water resources. These dyes have a long persistence in aquatic environments and can potentially exhibit harmful effects such as mutagenicity and carcinogenicity (H. Saroyan et al. 2019).

Remazol Brilliant Blue R, denoted hereafter as RBBR (C_22_H_16_N_2_Na_2_O_11_S_3_) (Fig. 1d), is an anionic (reactive) dye with anthraquinonic groups, being soluble in water. Anthraquinonic dyes are known for their vibrant and long-lasting colors, making them desirable for dyeing applications (Yeow et al. 2021).

Methylene blue, denoted hereafter as MB (C_16_H_18_ClN_3_S) (Fig. 1b) is a type of basic dye, extensively used in industries such as dyeing, printing, and pharmaceuticals. Cationic dyes are attracted to materials’ negatively charged surfaces, allowing them to form strong bonds (Kubra et al. 2021). However, it has been found to have detrimental effects on the central nervous system, leading to the development of various skin conditions. Consequently, the presence of MB in freshwater reservoirs is related with a potential hazard to both aquatic organisms and human beings (Dey et al. 2017).

Over the years, numerous approaches based on physical, chemical, and biological processes have been established to address the issue of dye removal from wastewater (Dai et al. 2018). A wide range of techniques have been employed to eliminate dye pollutants, including chemical oxidation (Saroyan et al. 2019), coagulation and flocculation (Ihaddaden et al. 2022), electrocoagulation (Saad et al. 2022; Taheri 2022), adsorption (Tolkou et al. 2023), membrane separation (Semiz 2020), ozonation (Ikhlaq et al. 2021), and fungal decolorization (Saad et al. 2022; Taheri 2022). Among these methods, adsorption is a highly popular, simple, and cost-effective technique for dye removal (Zhu et al. 2018).

Currently, there is a wide variety of commercially available adsorbents designed for various applications, such as zeolites (Ikhlaq et al. 2021), chitosan (Rostamian et al. 2022; Zeng et al. 2023), magnetic nanocomposites (Panda et al. 2021), graphene oxide (H. Saroyan et al. 2019), and activated carbon (Nizam et al. 2021). Activated carbon is highly effective in removing organic pollutants, and its properties, such as its porous texture and functional groups, are important for dye adsorption (Ndagijimana et al. 2021). However, the economic viability of activated carbon may be a limitation despite its extensive use. As a result, researchers have been investigating alternative sources of adsorbents, such as agricultural wastes that provide a cost-effective and sustainable solution for dye removal from wastewater (Zazycki et al. 2018). These agricultural wastes that have already been used as adsorbents are from shells (such as coconut (Tolkou et al. 2022), roots (*Alcea rosea* (Mahmoudabadi et al. 2019)), nuts (areca (Sukla Baidya and Kumar 2021)), fruit peels (i.e.. peels from banana (Mondal and Kar 2018), orange (Munagapati et al. 2019), watermelon (Krueger et al. 2019)), and leaves (cassava (Chairunnisa and Lisafitri 2020)). Some of them have been chemically treated to enhance their effectiveness, such as orange peels (Munagapati et al. 2019). Therefore, it is necessary to prepare more sustainable and cost-effective adsorbents using agricultural wastes with low cost.

Banana peels are often discarded in juice factories and fruit shops. Bananas are part of the *Musaceae* family, which consists of several hybrid varieties within the *Musa* genus (Hikal et al. 2022). Banana peels are low-cost materials and contain significant amounts of dietary fibers and phenolic compounds (Vu et al. 2018). Orange peels, which are numerous, inexpensive, and widely available materials abandoned at fresh fruit stalls and juice stores, may be employed as applied economic adsorbents for the removal of various contaminants from aqueous solutions, such as dyes and heavy metal ions. This type of peels contains cellulose, lignin, pectin (galacturonic acid), hemicelluloses, and chlorophyll pigments (Ayala et al. 2021). These constituents possess various functional groups, such as amide, carboxyl, and hydroxyl groups, which can play a significant role in the effective removal of dyes from wastewaters (Munagapati et al. 2019). Pomegranate peels are plentiful, cost-effective, and readily accessible materials derived from agricultural wastes. They have been effectively employed as affordable adsorbents for the separation of phenols, phenolic compounds, chromium(VI), nickel, and crude oil from simulated wastewater (Drinić et al. 2020; Ramadan et al. 2021). This low-cost material has proven its potential in efficiently removing these pollutants, showcasing its viability as a cost-effective solution for wastewater treatment (Saigl and Ahmed 2021).

The present study focuses on the removal of four different dyes (RR120, RB5, RBBR, and MB), representing both the anionic and cationic dye categories, from wastewaters by adsorption. As adsorbents, banana, orange, and pomegranate peels were used, washed and dried, and applied without further chemical modification. The aim of this study was to prepare natural adsorbents derived from fruit peels comprising environmental-friendly and affordable materials for the effective removal of dyes. In many studies, banana, orange, and pomegranate peels have been used as adsorbents for dye removal (Ben-Ali 2021; Daud et al. 2019; Kapoor et al. 2022; Michael-Igolima et al. 2023; Munagapati et al. 2018). However, in these studies, the materials were applied after chemical modification. In this work, the focus was on the development of cost-effective adsorbents due to their more economical and less invasive way of preparation. Therefore, the novelty of the present study is clear because these peels were prepared and used without further chemical modification, and in addition, the combination of these adsorbents used and the examined dye removal has not been studied, yet. The research examined the influence of various factors, initial pH value, adsorbent dosage, initial dye concentration, and contact time. The natural adsorbents used were analyzed for their structural and morphological properties using techniques such as BET, FTIR, and SEM/EDS. Kinetic and isotherm models were also analyzed to understand and evaluate the adsorption process, while thermodynamic and regeneration studies were conducted.

## Materials and methods

### Materials

Various locally available and low-cost materials, i.e., banana, orange, and pomegranate fruits, were collected from the local markets and used as tentative absorbents to remove dye from aqueous solutions. The peels of those fruits usually are thrown as wastes. Two (reactive) anionic dyes, which are commercially known as Reactive Red 120 (RR120) (dye content, ≥ 90%) and Reactive Black 5 (RB5) with a purity of ≥ 50% (supplied by Kahafix), one anthraquinone dye, i.e., Remazol Brilliant Blue R (RBBR) with 95% purity, as well as a cationic one known as Methylene Blue (MB) ≥ 95%, were purchased from Sigma-Aldrich-Merck KGaA, Darmstadt, Germany, and used as adsorbates. It is noted that the purity of the dyes was included in all calculations. The chemical structure and formula of these commercially available dyes are shown in Fig. 1. Stock dye aqueous solutions of 1000 mg/L were prepared by dissolving 1 g of dye in distilled water. The solution pH, where it was necessary, was adjusted by using HCl 37% v/v (supplied by Panreac) or NaOH (pellets, ACS reagent, ≥ 97.0%, supplied by Sigma-Aldrich). Finally, for the regeneration experiments, alkali solutions of 1.0 M NaOH were used.

### Preparation of natural adsorbents

Fruit peels were collected before being thrown away as wastes and processed using a green approach. Hence, a big amount of peels of banana, orange, and pomegranate were then extensively washed carefully with deionized water to remove any dirt or impurity. Selected peels are then air-dried at room temperature for 3 days before sieved and oven-dried at 100 °C (373 K) for 2 h. After drying, the collected fruit peels were grinded in small pieces by a domestic mixer into powder form (75–125 μm) in order to be used in adsorption experiments. The weight of the resulting powder of each natural adsorbent (Appendix 1) was recorded and then sealed in a plastic bag and stored in a cold room (Azamzam et al. 2022; Ben-Ali 2021; Shaharom 2019). At this point, it should be noted that the amount of sorbent produced was sufficient for all experiments and characterizations.

### Characterization techniques of adsorbents

Several characterization techniques were applied to study the surface of the adsorbents before and after the adsorption experiments. Among these techniques, scanning electron microscopy (SEM) (Jeol JSM-6390 LV, Japan scanning electron microscop)/ EDS, Fourier transform infrared spectroscopy (FT-IR, PerkinElmer, New York, NY, USA), and Brunauer, Emmett and Teller (BET) analysis software and X-ray diffraction (XRD) (Bruker D8 FOCUS, CuKα, *λ* = 0.154 nm) were applied. Characterization measurements were conducted in several samples, and the results were found to be repeatable and in agreement with all other non-shown measurements. Therefore, here are presented representative results.

### Analytical determinations of dye concentration

After each adsorption experiment, water samples were collected from the supernatant of each falcon tube, then filtered through a nylon membrane filter (0.45 μm) and stored for immediate determination. The initial and residual dye concentration was determined by corresponding the absorbance taken from a UV–Vis spectrophotometer (WTW Spectroflex 6100, Weilheim, Germany), to the standard curves of each dye, at different wavelengths depending on the dye. Thus, the relative *λ*_max_ were 515, 603, 593, and 664 nm for RR120, RB5, RBBR, and MB, respectively (Arya et al. 2020a; Kuang et al. 2020; Samarghandy et al. 2011; Travlou et al. 2013). For the repeatability of experiments and precision of measurements of the obtained results, the relevant experiments were repeated in triplicate, and in addition, the measurement of each sample was taken three times. Data were expressed as trace element mean values ± standard deviation.

### Adsorption experiments

The efficiency of banana, orange, and pomegranate peels as adsorbents for RR120, RB5, RBBR, and MB removal was investigated through a series of single-component experiments. Falcon tubes were used, each containing 10 mL of RB5 dye solution with varying initial concentrations needed. The temperature was kept constant throughout the experiments, and the mixture was stirred at 80 rpm using a Trayster overhead shaker and Loopster rotator. Throughout the experiments, various experimental factors were adjusted individually, while ensuring that all other parameters remained unchanged. These factors included pH levels (ranging from 2.0 to 9.0); initial RR120, RB5, RBBR, and MB dye concentrations (ranging from 5 to 1000 mg/L); adsorbent doses (ranging from 1 to 7 g/L, according to preliminary experiments and achieved by adding 0.01–0.07 g to the working volume of 10 mL); and contact time (ranging from 5 to 1440 min (24 h)). Following the adsorption process, samples were collected from the tubes and passed through a 0.45-μm nylon filter. The filtrate was then used for subsequent analyses. The average values of triplicate experiments were used as experimental data points. The percentage removal (*R* (%)) of RR120, RB5, RBBR, and MB dyes was calculated from the following equation (Eq. 1):

1 $$R \left(\mathrm{\%}\right)= \left(\frac{{C}_{0}-{C}_{f}}{{C}_{0}}\right)\times 100\mathrm{\%}$$

where *C*_0_ represents the initial RR120, RB5, RBBR, and MB concentrations in mg/L and *C*_*f*_ represents the final dye concentrations after the experimental process, calculated in mg/L.

To determine the adsorption capacity of the adsorbents, *Q*_*e*_ in mg/g was calculated using Eq. 2 as follows:

2 $${Q}_{e} = \frac{({C}_{0}-{C}_{e})\times V}{m}$$

where *C*_*e*_ indicates the RR120, RB5, RBBR, and MB concentration (mg/L) at equilibrium, *V* indicates the volume of dye solutions (L), and *m* indicates the mass of each adsorbent added (g).

#### Equilibrium experiments

In the isothermal experiments, a fixed amount of banana, orange, and pomegranate peel adsorbents (in g) was added to 15-mL falcon tubes containing 10 mL of RR120, RB5, RBBR, and MB dye solutions. The concentrations of each dye solution ranged from 5 to 1000 mg/L, by keeping constant the initial pH value and the temperature, at 24 h as contact time. The data collected from these experiments were analyzed using the commonly used and easiest to apply experimentally Langmuir and Freundlich isotherm models, as the Langmuir model is the foundation upon which much of adsorption theory is built and therefore provides a useful conceptual basis for understanding the process (Kalam et al. 2021). The Langmuir and Freundlich models are expressed mathematically by Eqs. 3 and 4, respectively.

3 $${Q}_{e}= \frac{{Q}_{m}{K}_{L}{C}_{e}}{1+{K}_{L}{C}_{e}}$$

4 $${Q}_{e} = {K}_{F}{C}_{e}^{1/n}$$

where *Q*_*e*_ represents the equilibrium concentrations of the adsorbates in the solid phase relative to the equilibrium concentrations in the liquid phase, measured in mg/g. *Q*_*m*_ represents the maximum adsorption capacity, which depicts the theoretical capacity of a monolayer, measured in mg/g. *K*_*L*_ represents the energy associated with the adsorption of RR120, RB5, RBBR, and MB dyes, measured in L/mg. *K*_*F*_ ((mg/g)∙(L/mg)^1/*n*^) constant related to the adsorption capacity, and 1/*n* constant associated with the adsorption intensity or the surface heterogeneity.

According to the Langmuir theory, adsorption occurs when the adsorbate molecules form monolayer on the surface of the adsorbent, without interacting with each other. This theory also supposes that the adsorbent has a limited adsorption capacity (*Q*_*m*_), representing the maximum amount of adsorbate that can be adsorbed under equilibrium conditions.

Otherwise, the Freundlich model establishes a relationship between the equilibrium concentrations of dyes (mg/L) and the adsorption capacity of the adsorbent, represented by *Q*_*e*_ (mg/g). Freundlich model is an empirical model based on adsorption on a heterogeneous surface that is valid only up to a certain concentration and assumes the formation of multilayer and heterogeneous systems (Arami et al. 2005; Jaroniec 1975).

#### Kinetic experiments

The dye removal capacity of peels adsorbents also depends on the contact time. Kinetic experiments were carried out at optimum pH values, constant temperature, and different contact times (0–210 min). The kinetic study investigated the adsorption of RR120, RB5, RBBR, and MB dyes using both the pseudo-first-order (PFO) (Eq. 5) and pseudo-second-order (PSO) (Eq. 6) models. These models are mainly applied in many adsorption studies (Revellame et al. 2020). The obtained kinetic parameters were utilized to estimate the adsorption process and identify suitable rate expressions for potential reaction mechanisms. These analyses were conducted to enhance the understanding of banana, orange, and pomegranate peels onto dye adsorption.

5 $${Q}_{t}= {Q}_{e}\left(1-{e}^{-{k}_{1}t}\right)$$

6 $${Q}_{t}=\frac{{k}_{2}{Q}_{e}^{2}{\text{t}}}{1+{k}_{2}{Q}_{e}t}$$

where parameters *Q*_*t*_ and *Q*_*e*_ represent the amount of RR120, RB5, RBBR, and MB dyes adsorbed (in mg/g) at a given time *t* (min) and at equilibrium, respectively. *k*_1_ (L/min) and *k*_2_ (g/mg∙min) constants correspond to the rates of adsorption for the pseudo-first-order (PFO) and pseudo-second-order (PSO) models, respectively. *t* denotes the duration of the contact time in minutes.

#### Thermodynamics

Temperature is a variable that affects the adsorption process, and the relative effect on dye adsorption was observed at different temperatures, such as 298, 308, 318, and 338 K, applying the optimum dosage and pH value for 90 min as contact time. The change of Gibbs free energy (*ΔG°*, kJ/mol)*,* enthalpy (*ΔH°*, kJ/mol), and entropy (*ΔS°*, kJ/mol·K) is considered to thermodynamically evaluate the adsorption process and tentatively characterize its nature. Consequently, to calculate the thermodynamic parameters, four different temperatures (298, 308, 318, and 338 K) were conducted and the following equations (Smith 1916–2009, 1959) were applied:

7 $${K}_{c}=\frac{{C}_{s}}{{C}_{e}}$$

8 $$\Delta G^\circ =-RT{\text{ln}}\left({K}_{c}\right)$$

9 $$\Delta G^\circ =\Delta H^\circ -{\text{T}}\Delta S^\circ$$

10 $${{\text{ln}}(K}_{{\text{c}}})=\left(-\frac{\Delta H^\circ }{R}\right)+\frac{\Delta S^\circ }{R}$$

where *K*_C_ is the thermodynamic constant, *R* is the universal gas constant (8.314 J/mol·K), and *T* is the temperature (K), while *C*_s_ (mg/L) is the amount adsorbed on solid at equilibrium and* C*_*e*_ indicates the RR120, RB5, RBBR, and MB concentration (mg/L) at equilibrium (Kuang et al. 2020; Munagapati et al. 2019)*. ΔG°* was given from Eq. 8, and the values of *ΔH°* and *ΔS°* were calculated from the slope and intercept of the plot of ln(*K*_*c*_) versus 1/*T* (Eq. 10).

## Results and discussion

### Characterizations

#### Physical properties

BET analysis was carried out to determine the surface area (m^2^/g), pore diameter (nm), and total pore volume (cm^3^/g) of the adsorbents, by using Barrett-Joyner-Halenda (BJH) analysis. The relative values are listed in Table 1, and as shown, banana peels have a larger surface area and total pore volume (73.2 m^2^/g and 0.231 cm^3^/g, respectively) relative to that of orange peels (63.2 m^2^/g and 0.195 cm^3^/g) and of pomegranate peels (35.9 m^2^/g and 0.109 cm^3^/g). On the other hand, regarding the diameter of the mean pores, it was found that all adsorbent materials present similar values, i.e., 1.613, 1.618, and 1.615 nm.

**Table 1:** Physical properties of banana, orange, and pomegranate peels

Parameters	Banana peels	Orange peels	Pomegranate peels
BET surface area, *S*_BET_ (m^2^/g)	73.2	63.2	35.9
Mean pore diameter (nm)	1.613	1.618	1.615
Total pore volume, *V*_*T*_ (cm^3^/g)	0.231	0.195	0.109

#### Scanning electron microscopy and EDS analysis

**Fig. 2:**
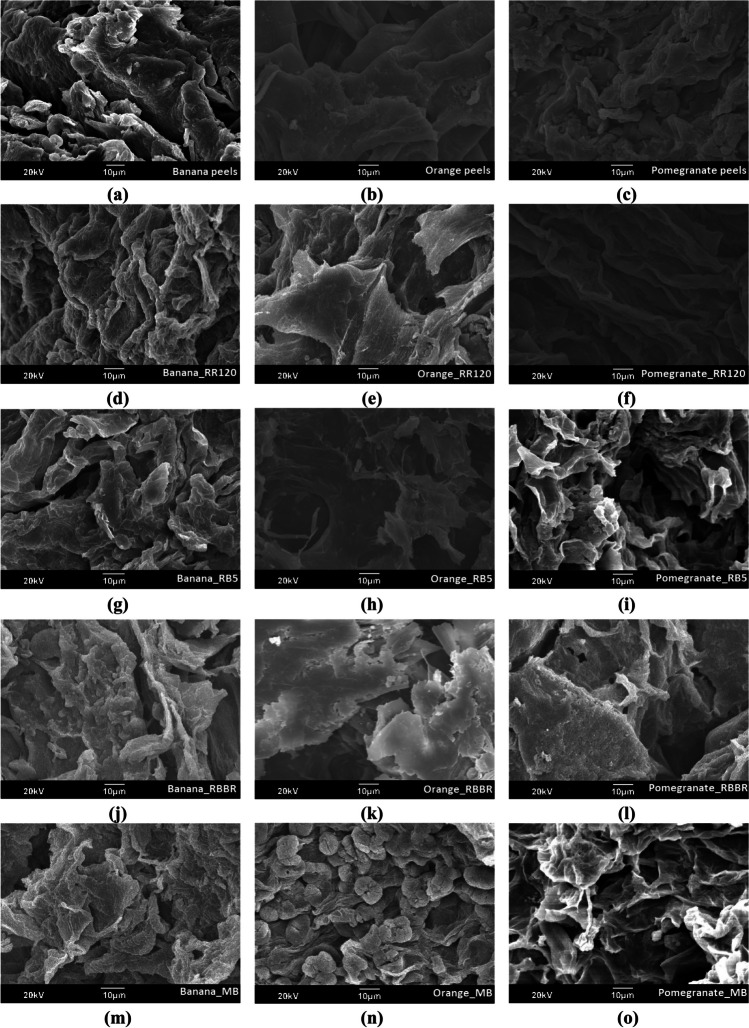
SEM images of **a** banana, **b** orange, and **c** pomegranate peels, before and after dye adsorption: **d** banana_RR120; **e** orange_RR120; **f** pomegranate_RR120; **g** banana_RB5; **h** orange_ RB5; **i** pomegranate_RB5; **j** banana_RBBR; **k** orange_RBBR; **l** pomegranate_RBBR; **m** banana_MB; **n** orange_MB; and **o** pomegranate_MB

The SEM micrographs of banana, orange, and pomegranate peels before and after the adsorption process were taken and illustrated in Fig. 2. As shown, banana peels (Fig. 2a) and pomegranate peels (Fig. 2b) exhibited highly heterogeneous and irregular porous structure which supports the adsorption of RR120 (Fig. 2d–f), RB5 (Fig. 2g–i), RBBR (Fig. 2j–l), and MB (Fig. 2m–o). After adsorption, peels appear to have rough surface with wider pores (Munagapati et al. 2018), as the pores were packed with dyes (Arami et al. 2005). Moreover, the SEM image of orange peels (Fig. 2b) shows a cleaner and smoother surface and less porous structure, compared to that of banana and pomegranate peels; this may exhibit less adsorption capacity due to the reduced number of active adsorption sites available for dye binding (Michael-Igolima et al. 2023). After adsorption of MB on orange peels (Fig. 2n), a change in microstructure and morphology of the surface is observed, in comparison with the adsorption of RR120 (Fig. 2e), RB5 (Fig. 2h), and RBBR (Fig. 2k). Similar structural divergences have been reported in previous studies (Stavrinou et al. 2018). This gives interest for the continuation of the research and the correlation of these findings with the adsorption capacity of these materials.

**Fig. 3:**
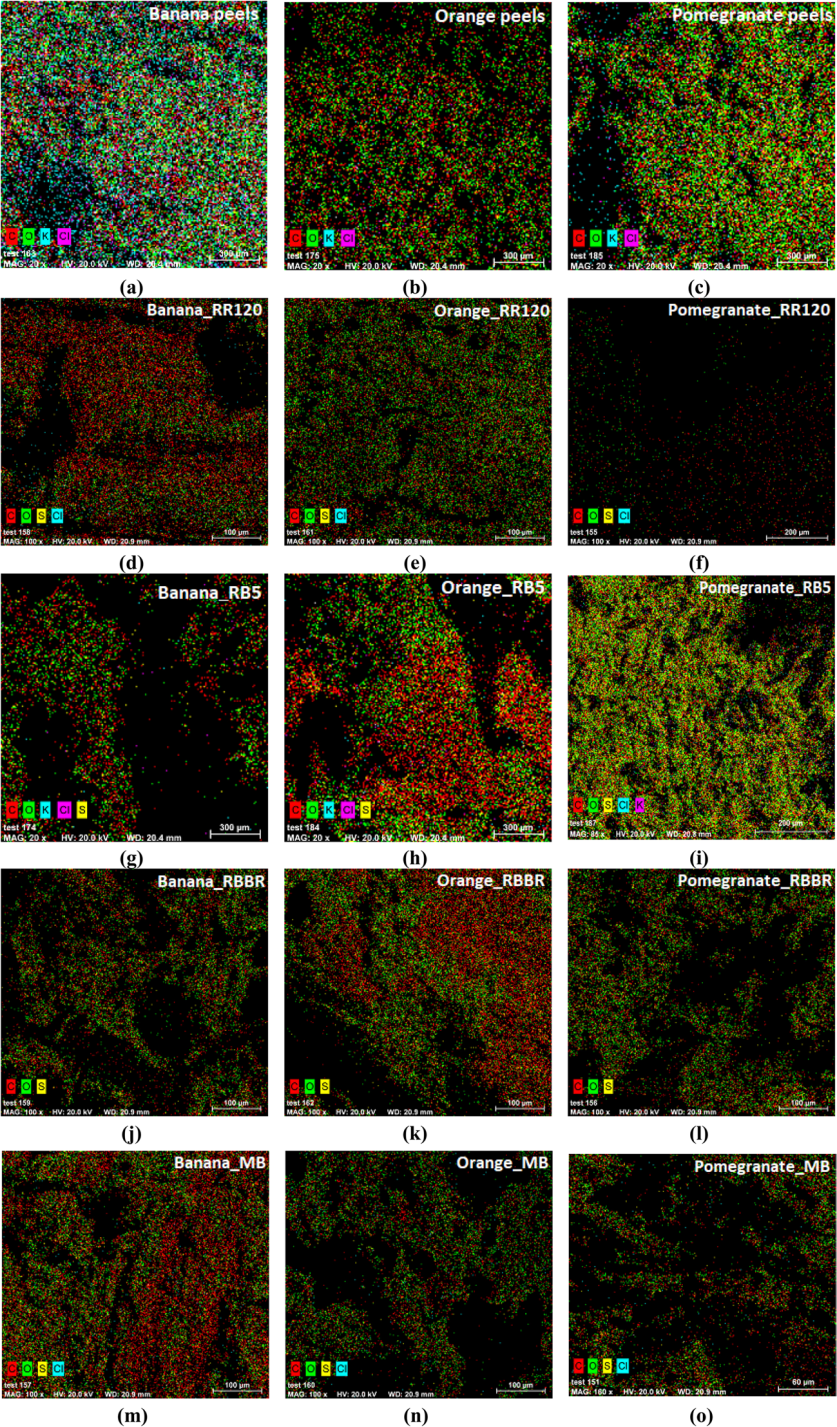
Adsorbed dye distribution maps on **a** banana, **b** orange, and **c** pomegranate peels, before and after adsorption: **d** banana_RR120; **e** orange_RR120; **f** pomegranate_RR120; **g** banana_RB5; **h** orange_ RB5; **i** pomegranate_RB5; **j** banana_RBBR; **k** orange_RBBR; **l** pomegranate_RBBR; **m** banana_MB; **n** orange_MB; and **o** pomegranate_MB, by SEM/EDS analysis

Furthermore, EDS analysis of adsorbents before and after dye adsorption is conducted, and the results are presented in Fig. 3 and Table 2. EDS can be used to determine which chemical elements are present in a sample and also to estimate their relative abundance. In addition, EDS analysis after adsorption was determined in order to check if there is any modification in the percentage of elements on the surface of the materials, which possibly actively participating in the removal of the dye. As shown, C, Cl, K, and O elements were recorded on the surface of banana, orange, and pomegranate peels (46.45, 0.97, 3.46, and 49.13%; 56.01, 0.19, 0.88, and 42.92%; and 49.77, 0.27, 1.11, and 48.85%, respectively), confirming the homogeneous distribution of these elements on the surface. After the adsorption, a greater percentage of carbon content and the appearance of sulfur are observed, which are possibly due to the additional C and S that resulted from the structure of the adsorbed dyes (see Fig. 1). After adsorption, a decrease or in some cases disappearance of the percentage of K is observed, possibly related to the removal and binding of the dye especially in the case of banana peels as the 3.46% before adsorption, set to almost zero (or values closed to that) after adsorption of RR120, RBBR, and MB and to 0.1% of RB5.

**Table 2:** Elemental composition of adsorbents before and after dye adsorption

% (w/w)	Banana	Banana/RR120	Banana/RB5	Banana/RBBR	Banana/MB
Carbon	46.45	60.79	54.92	68.23	66.29
Chlorine	0.96	0.11	0.14	-	0.18
Potassium	3.46	-	0.10	-	-
Oxygen	49.13	39.02	44.36	31.47	33.09
Sulfur	-	0.09	0.48	0.30	0.44
% (w/w)	Orange	Orange/RR120	Orange/RB5	Orange/RBBR	Orange/MB
Carbon	56.01	57.66	45.36	62.22	59.95
Chlorine	0.19	0.07	0.69	-	0.06
Potassium	0.88	-	1.00	-	-
Oxygen	42.92	42.08	52.37	37.29	39.85
Sulfur	-	0.19	0.58	0.49	0.14
% (w/w)	Pomegranate	Pomegranate/RR120	Pomegranate/RB5	Pomegranate/RBBR	Pomegranate/MB
Carbon	49.77	77.28	51.34	51.40	59.83
Chlorine	0.27	0.02	0.01	-	0.02
Potassium	1.11	-	-	-	-
Oxygen	48.85	22.45	48.46	48.35	39.75
Sulfur	-	0.25	0.20	0.25	0.39

#### FTIR analysis

**Fig. 4:**
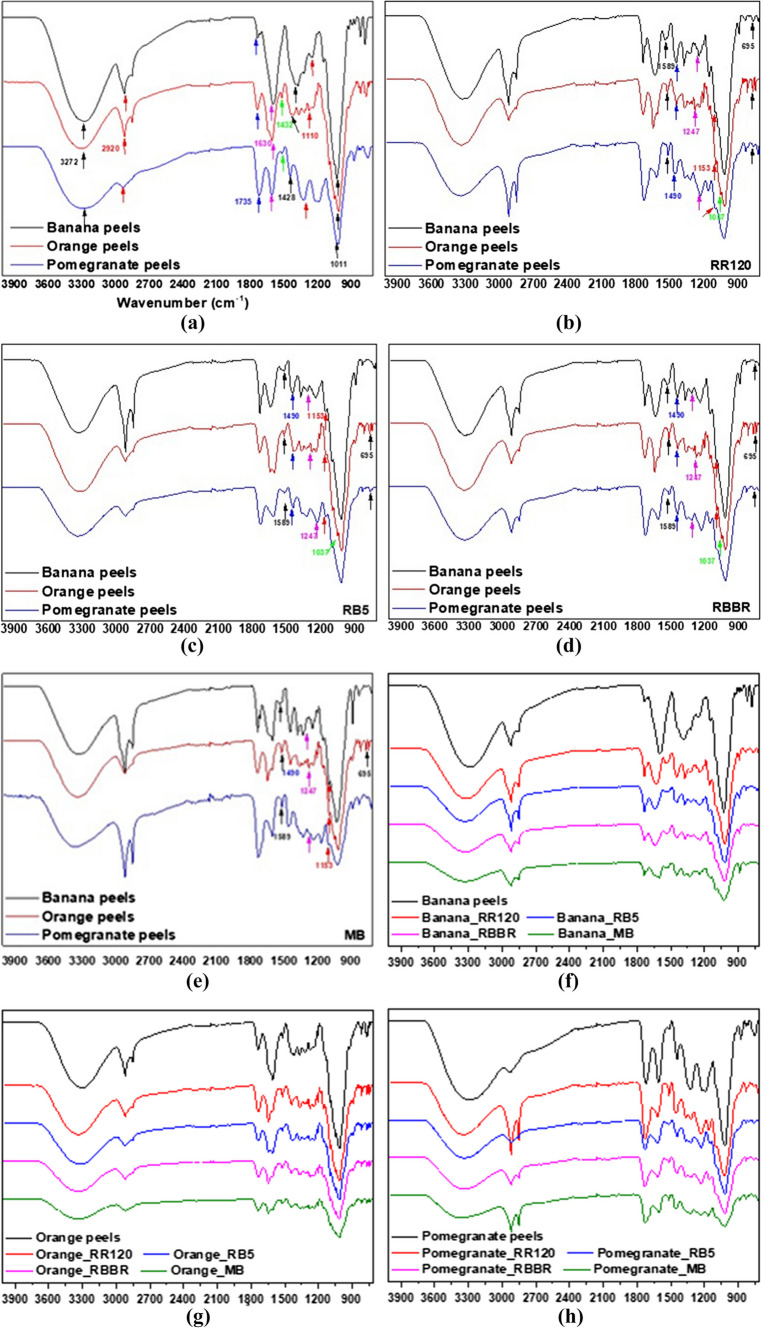
FTIR spectra of banana, orange, and pomegranate peels before and after dye adsorption

The FTIR spectra of banana, orange, and pomegranate peels before and after adsorption of RR120, RB5, RBBR, and MB, in the range of 4000–400 cm^−1^, were determined and compared to get information about the potential interactions between the adsorbent and each dye separately. These comparative spectra are shown in details in Fig. 4. Specifically, in Fig. 4a, the three adsorbents are compared in terms of their peaks in the FTIR spectrum before the adsorption of the dyes, while in Fig. 4b–e, the absorptions after the adsorption of RR120, RB5, RBBR, and MB, respectively, are presented and compared. In addition, Fig. 4f–h compare the effect of banana, orange, and pomegranate peels separately in this four dyes. The widespread band presented in all diagrams, around 3272 cm^−1^ matching to the stretching vibration of O–H groups and amine (–NH) functional groups (Al-Tabakha et al. 2021). The peak at 2920 cm^−1^ is accredited to aliphatic acids C–H stretching vibrations. The peak appeared at 1735 cm^−1^ may be due to the stretching vibrations of C = O due to the bonds of carboxylic groups (–COOH, –COOCH_3_) (Yang et al. 2007). The peaks at around 1630 and 1590 cm^−1^ correspond to skeletal aromatic vibrations of C = C in lignin (Gonultas and Candan 2018). Furthermore, the peak at 1432 cm^−1^ is attributed to phenol –COO– symmetric vibrations. Moreover, the peak at 1100 cm^−1^ may be attributed to the primary OH group in lignin or hemicellulose, regarding the composition of the peels (Mahrous et al. 2022). Additionally, the band at 1428 cm^−1^ is assigned to the C − H bending vibration, which forms the basic structure of lignocellulosic material, and the stretching at around 1011 cm^−1^ could confirm the existence of C–OH and C–OR groups found in carbohydrates (Michael-Igolima et al. 2023).

In addition, several other peaks that appeared after the adsorption are characteristic due to the structure of the dyes. Hence, at 1589 cm^−1^, the peak is relative to N − H bending vibrations of amine, at 1490 cm^−1^ relative to N = N stretching vibrations of azide; at 1247 cm^−1^, there is a peak regarding the stretching vibration of –S = O group and at 1153 cm^−1^ for stretching vibration of the –C − N. Moreover, the peak at 1028 cm^−1^ is characteristic for the stretching vibrations of alcoholic C–O, at 828 cm^−1^ for the aromatic C–H bending vibrations, and at 695 cm^−1^ may attributed to –S–O stretching (Munagapati et al. 2019). Consequently, the presence of hydroxyl and amine groups on the adsorbent surface is showing.

Several bands presented changes, i.e., at 1630 cm^−1^ and at 2928 cm^−1^, after adsorption of dyes. In addition, especially for banana and pomegranate peels, there is a new peak appeared that is not found in spectrums before adsorption. In addition, at 1476, 836, and 689 cm^−1^, new peaks appeared (Fig. 4b) indicating the formation of bonds between the peels and RR120 dye molecules (Munagapati et al. 2019). Therefore, it can be concluded that the hydroxyl and amine functional groups may play an important role in the adsorption of RR120 on the surface of the peels (Munagapati et al. 2019). Moreover, the spectra of the RB5 dye (Fig. 4c) showed similar characteristics and small changes in their positions and intensity occurred. These results specified the involvement of several functional groups (hydroxyl, amine, and carboxyl) in the adsorption of RB5 on the surface of the peels possibly due to weak electrostatic interactions or Van der Waals forces (Kapoor et al. 2022; Munagapati et al. 2018).

**Fig. 5:**
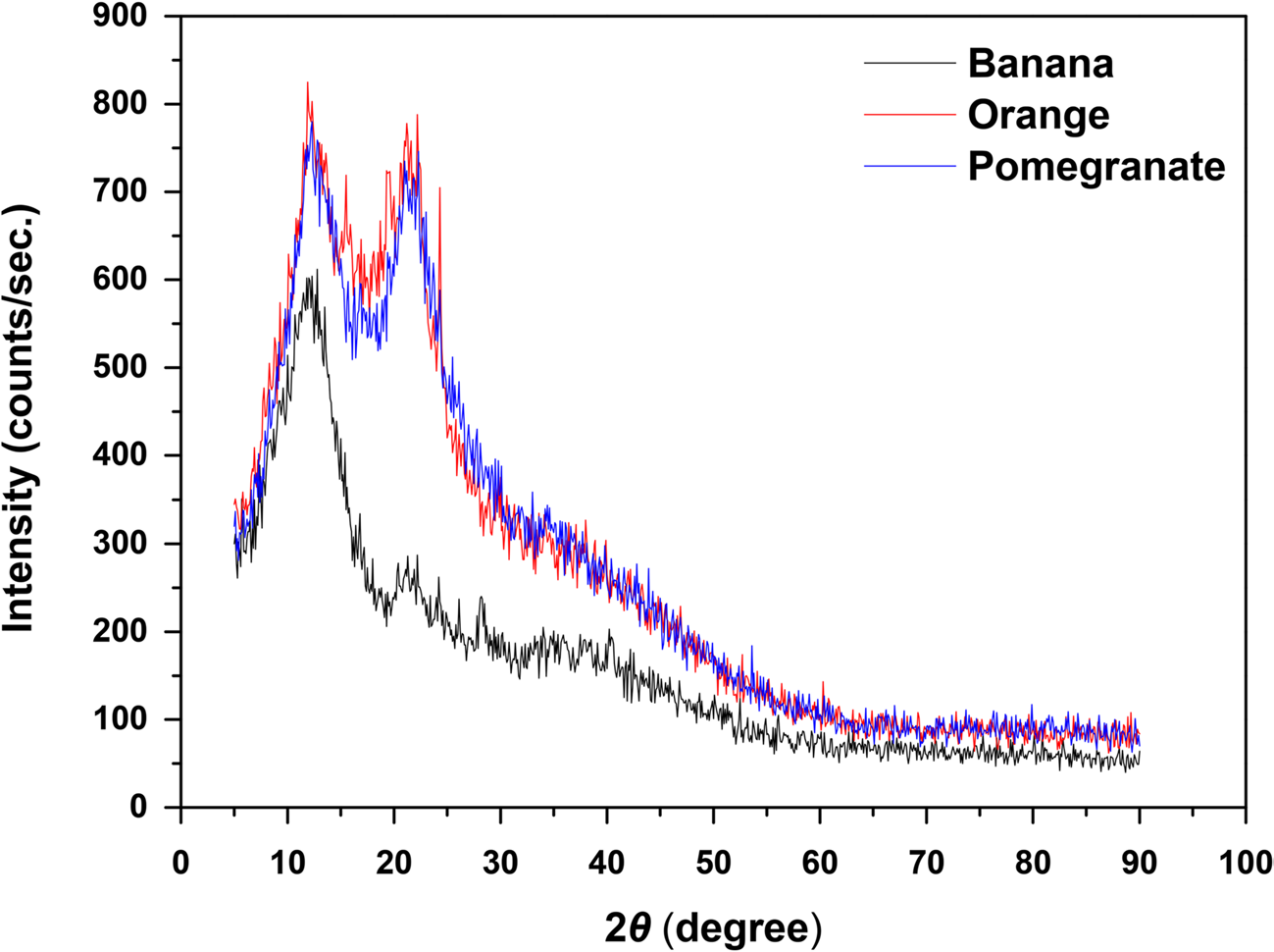
XRD patterns of banana, orange, and pomegranate peels

In addition, in Fig. 5d, at 1037 cm^−1^, the S = O stretching vibration is shown from sulfonic (–SO_3_^−^) groups (Sangar et al. 2019) that exist at RBBR structure (Tsoutsa et al. 2023). Some peaks in the spectra of the peels disappeared, decreased, or shifted after adsorption, possibly due to the adsorption of RBBR dye on their surface (Ahmad et al. 2020). Specifically, the peak at 1037 cm^−1^, due to S = O for –SO_3_^−^, particularly when orange peels are used as adsorbent, appears and was not presented before.

Finally, the FTIR spectrum of banana, orange, and pomegranate peels after MB dye adsorption (Fig. 4e) did not show much difference from the spectrum before adsorption. In addition to this, some small shifts in the peaks can be detected, which may provide evidence that the acidic functional groups, such as –COOH, are responsible for the binding of MB cationic dye molecules (Jawad et al. 2021).

#### XRD analysis

The diffraction of X-ray (XRD) was used in this study to further validate the structure of peels. Figure 5 shows the patterns of banana, orange, and pomegranate peels. All the samples have a broad peak centered at 2*θ* = 14° which corresponds to the (101), at 2*θ* = 17° to (111), and at 2*θ* = 23° to (002) carbon reflection (less intense regarding banana peels). These peaks can be attributed to the Miller indices of the crystalline cellulose (JCPDS #03–0829) (Li et al. 2018; Padmanabhan et al. 2022). As expected for organic adsorbents, banana, orange, and pomegranate peels have an amorphous structure as the broad peak in the 2*θ* 20–25^ο^ range is indicative of the amorphous carbon present in such adsorbents (Çatlıoğlu et al. 2021; Hashem et al. 2023; Tadesse et al. 2023).

### Batch adsorption experiments

#### Effect of adsorbent’s dose

**Fig. 6:**
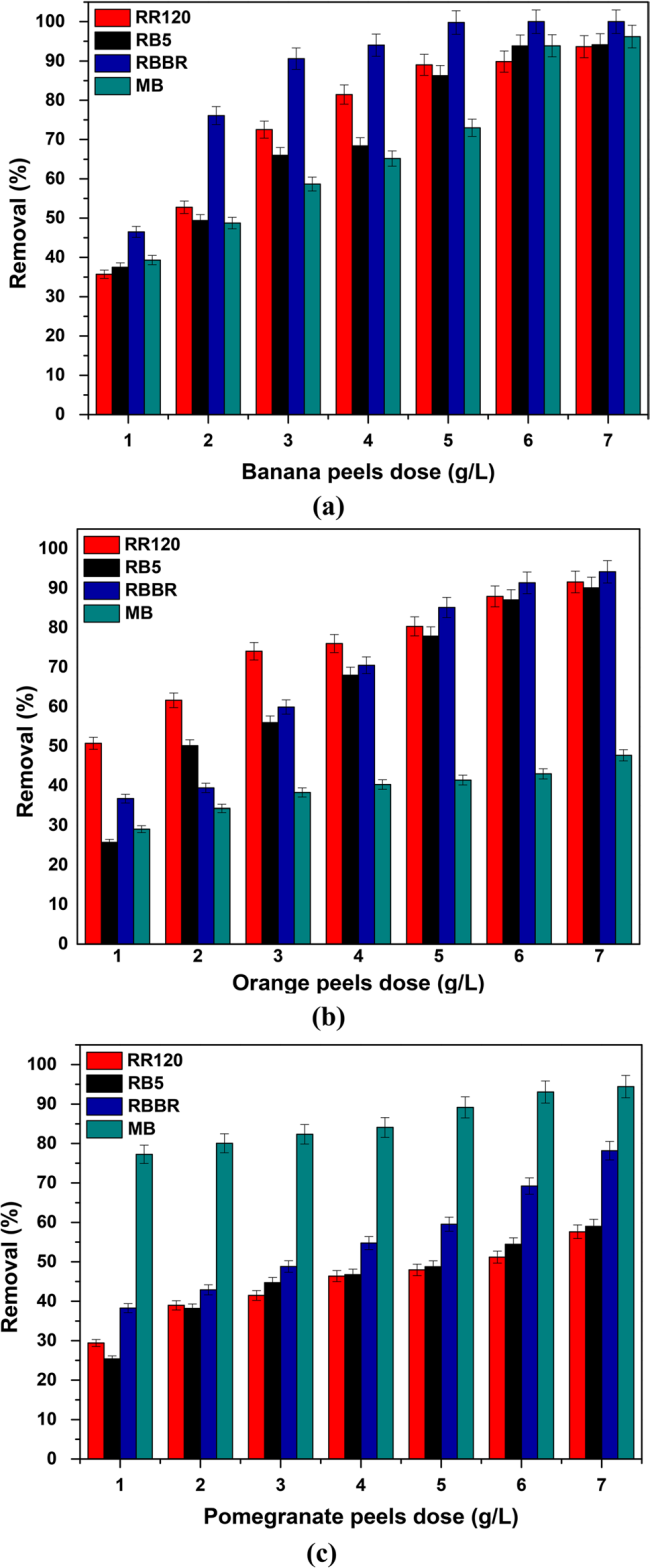
Effect of natural adsorbents’ dose on dye removal with **a** banana, **b** orange, and **c** pomegranate peels; *C*_0_ 100 mg/L, pH 2.0 ± 0.1, dose 1.0–7.0 g/L, *T* = 298 K, and contact time 24 h

Batch experiments were conducted to investigate the prospect of banana, orange, and pomegranate peels for the effective removal of RR120, RB5, RBBR, and MB dyes from wastewaters. In the experiments carried out, the initial concentration of each dye was 100 mg/L, at an indicative pH of 2.0 according to the literature (Devi and Prabhakar 2020; Mengelizadeh and Pourzamani 2020; Tsoutsa et al. 2023), a temperature of 298 K, and a contact time of 24 h, with the addition of different doses of the natural adsorbents in the range of 1.0–7.0 g/L. The relevant results are shown in Fig. 6, and as expected, in all cases, the dye removal rate increased as the dosage increased and more adsorption sites appeared on the adsorbent surface.

More specifically, when banana peels were used as adsorbent, as shown in Fig. 6a, 4.0 g/L was required to remove 80% of RR120 dye, a rate that increased to 90% with 6.0 g/L and to 94% with 7.0 g /L. In addition, for the removal of RB5, the 86% was removed by 5.0 g/L and 94% by 6.0 g/L. In the case of RBBR, by applying 3.0 g/L banana peels, the 90% of dye was removed, reaching the 100% with 5.0 g/L. Finally, for MB with 6.0 g/L, the relative rate reached 94%. In Fig. 6b, the effect of orange peel dosage is illustrated, and as depicted, in general, this adsorbent is less effective than banana peels. For instance, with 7.0 g/L of the adsorbent, the relative removal rates reached up to 92, 90, 94, and only 48% for RR120, RB5, RBBR, and MB, respectively.

However, in the case of using pomegranate peels as adsorbent (Fig. 6c), when applied to RR120, RB5, and RBBR dyes, the removal rates were found to be low, i.e., 58, 59, and 75%, respectively, even with 7.0 g/L. On the other hand, in the case of MB, pomegranate peels proved to be very effective even at lower doses, indicating that even fewer active sites on the adsorbent surface are capable of removing MB. This will be of interest in the continuation to be related to the pH_PZC_ of the material. Particularly, with the addition of only 2.0 g/L, 80% of the initial dye concentration was removed, a percentage that increased to 93% with 6.0 g/L and to 95% with 7.0 g/L.

**Fig. 7:**
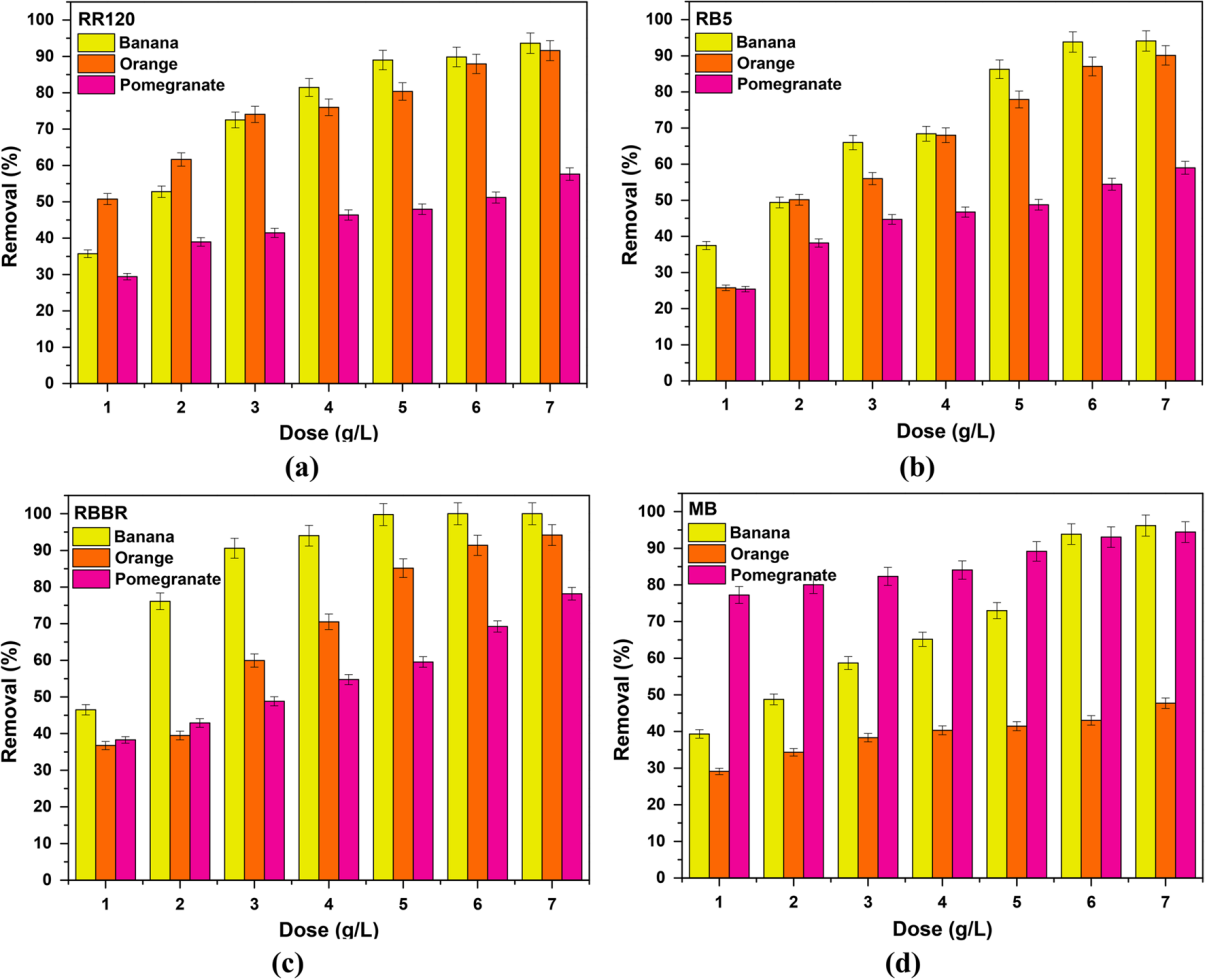
Comparison of natural adsorbents on **a** RR120, **b** RB5, **c** RBBR, **d** MB removal. *C*_0_ 100 mg/L, pH 2.0 ± 0.1, dose 1.0–7.0 g/L, *T* = 298 K, and contact time 24 h

Moreover, according to Fig. 7, where the effect of the adsorbents’ dose on each dye is analyzed separately and at the same time these materials are compared in terms of their effectiveness, the relative efficiency order emerged as follows:

- RR120: banana > orange > pomegranate peels (Fig. 7a)
- RB5: banana > orange > pomegranate peels (Fig. 7b)
- RBBR: banana > orange > pomegranate peels (Fig. 7c)
- MB: pomegranate > banana > orange peels (Fig. 7d)

Taking into account all the above that appeared from the analysis of Figs. 6 and 7, for the further study of the effectiveness of the adsorbent materials, the following doses were chosen:

- Banana peels: 6.0 g/L for RR120; 6.0 g/L for RB5; 5.0 g/L for RBBR; and 4.0 g/L for MB.
- Orange peels: 6.0 g/L for RR120, RB5, RBBR, and MB.
- Pomegranate peels: 6.0 g/L for RR120, RB5, RBBR, and MB.

A cost analysis was conducted for the preparation of peels as adsorbents (Appendix 1). As shown, for using 6.0 g/L as indicative dosage, the cost was around 0.196 €/L for banana peels (final working volume is 10 mL = 0.0020 €/experiment), 0.129 €/L (0.0013 €/experiment) for orange peels, and 0.277 €/L (0.0028 €/experiment) for pomegranate peels. Hence, these are low-cost adsorbents prepared from waste fruit peels.

#### Effect of initial solution pH

**Fig. 8:**
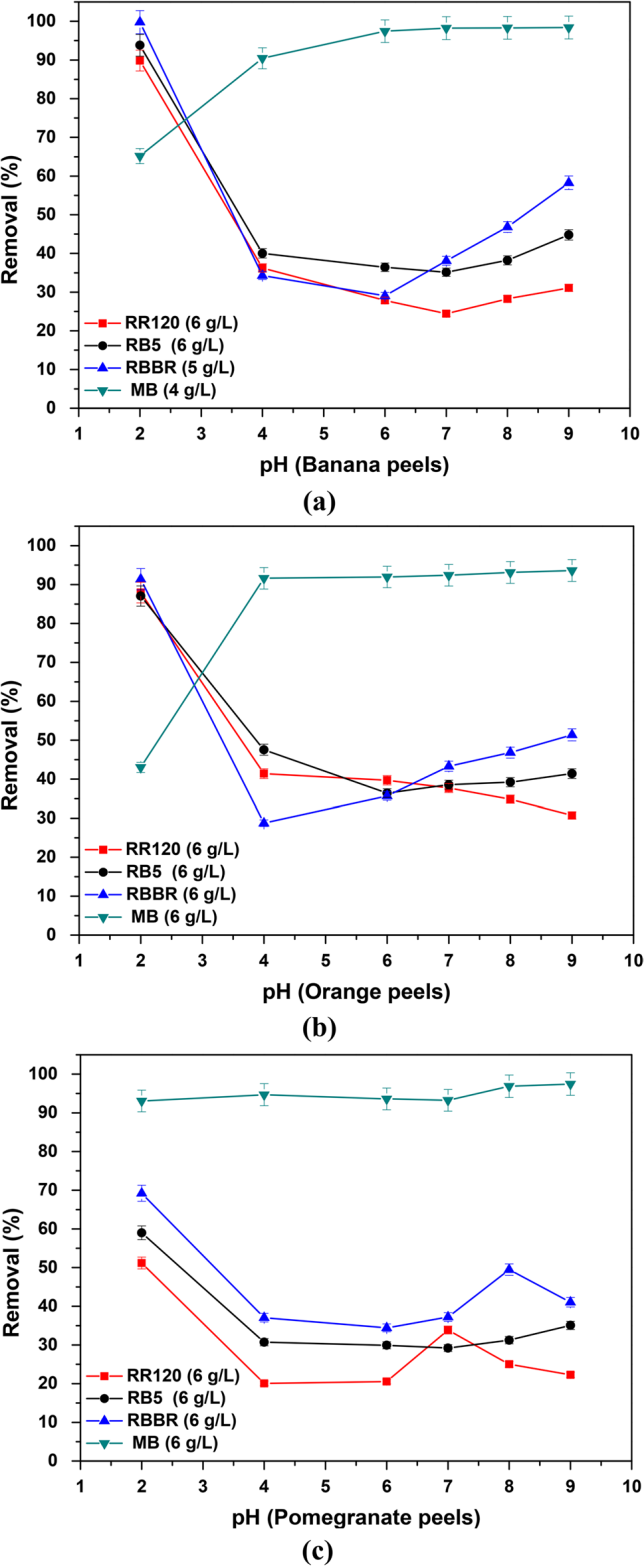
Effect of initial solution pH on the adsorption of dyes removal with **a** banana, **b** orange, and **c** pomegranate peels; *C*_0_ 100 mg/L, pH 2.0–9.0 ± 0.1, *T* = 298 K, contact time 24 h

One of the most important factors that can affect the adsorption performance is the effect of the initial solution pH. Therefore, the effect of pH on the adsorption capacity of RR120, RB5, RBBR, and MB dyes onto banana (Fig. 8a), orange (Fig. 8b), and pomegranate (Fig. 8c) peels was studied in various pH conditions in the range of 2.0 to 9.0 at 298 K, at a constant initial dye concentration of 100 mg/L, and adsorbent dosage according to the results achieved in the previous section for each dye and adsorbent. The obtained results are shown in Fig. 8 and will be further analyzed below.

**Fig. 9:**
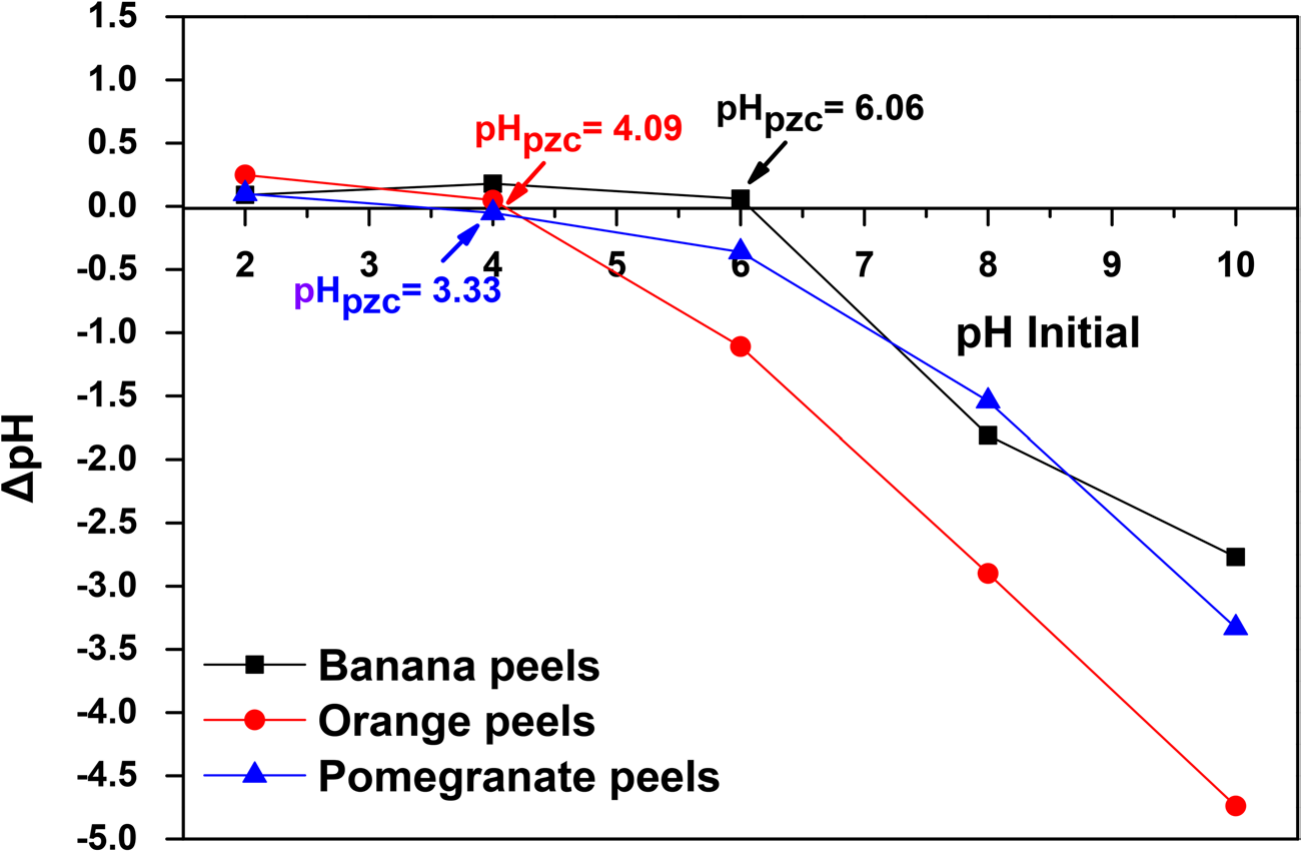
Determination of pH_pzc_ of natural adsorbents using pH drift method (Tai et al. 2020)

In addition, the point of zero charge (pH_pzc_) of each material is a very important parameter for obtaining information about the surface charge of the adsorbent, as it is the value at which the surface charge of the material becomes neutral. At solution pH below pH_pzc_, the adsorbent surface is positively charged and can attract negatively charged ions, while at solution pH values greater than the pH_pzc_, the surface is negatively charged and attracts cations. In this study, pH_pzc_ was determined by measuring it over a pH range of 2.0–10.0 ± 0.1, according to pH drift method (Tai et al. 2020). Hence, as shown in Fig. 9, the calculated pH_pzc_ values were 6.06, 4.09, and 3.33 for banana, orange, and pomegranate peels, respectively.

Based on the above, the effect of pH is further discussed for the removal of these dyes, taking also in account the results obtained from Fig. 8. Hence, regarding the adsorption of RR120 dye, in all materials, it is shown that it is significantly dependent on the pH of the solution. In general, the removal percentage of RR120 decreased from 90 to 31% by using banana peels, from 88 to 31% by orange peels, and from 51 to 22% by using pomegranate peels, with increasing pH solution from 2.0 to 9.0. Thus, the maximum removal rate was reached at 2.0; therefore, this pH was chosen for further adsorption experiments. According to literature, RR120 has water-soluble sulphonate (R-SO_3_^−^) groups (Fig. 1a) (Munagapati et al. 2019), leaving the dye molecule with a net negative charge, as shown in the following Reaction (11):

11 $$RR120-{SO}_{3}Na \to RR120-{SO}_{{3}^{-}}+{Na}^{+} R.$$

By comparing the pH_pzc_ values of banana (6.06), orange (4.09), and pomegranate (3.33) peels with the optimum pH of RR120 solution, an explanation can be given, as the positive surface charge of the peels attracts the negative charged RR120 and enhances uptake. However, at higher pH values, the abundance of hydroxylic ions competes with RR120 and ionic repulsions between the negatively charged surface and the RR120 molecule resulting in lower RR120 adsorption, as the surface of all peels lacks of exchangeable anions at higher pH values (Bazrafshan et al. 2013; Munagapati et al. 2019), due to the loss of protons in a basic solution.

In the case of RB5 dye, the percentage removal was also decreased with the increase of pH. Particularly, from the high removal rate of 94% at pH 2.0 to 45% at pH 9.0, when the banana peels were used as the adsorbent (Fig. 8a), this is evident. Similarly, to banana peels, when orange peels (Fig. 8b) or pomegranate peels (Fig. 8c) are used, there is also a decrease in dye removal rates as pH increases. RB5 is also a reactive dye with sulphonate (R-SO_3_^−^) groups (Fig. 1c), and for this reason, an identical reaction takes place (Reaction (12)):

12 $$RB5-{SO}_{3}Na\to RB5-{SO}_{{3}^{-}}+{Na}^{+} R.$$

At higher pH, the electrostatic repulsion between the negatively charged surface of the adsorbents and the anionic ions of the RB5 dye causes a significant drop in dye removal. Therefore, the maximum dye removal by adsorption on banana, orange, and pomegranate peels was achieved at pH 2.0, at which the surface of the adsorbents and the functional groups of the dye behave in a relative manner. Thus, at acidic pH 2.0 (a value lower than any pH_pzc_ determined), the surfaces of the materials are positively charged, and with the sulfonic group (–SO_3_^−^) of the surface of RB5, a strong electrostatic attraction is expected. On the other hand, the lower adsorption capacity in the alkaline condition (pH 9.0) is due to the competition between hydroxyl ions (OH^−^) and the negatively charged dye ions for the adsorption sites (Devi and Prabhakar 2020; Mengelizadeh and Pourzamani 2020; Tsoutsa et al. 2023).

RBBR dye is also a reactive anthraquinone dye with sulphonate (R-SO_3_^−^) groups (Fig. 1d), which upon its dissolution in water behaves in the same way (Reaction (13)):

13 $$RBBR-{SO}_{3}Na\to RBBR-{SO}_{{3}^{-}}+{Na}^{+} R.$$

Similar to previous results in the literature (Arya et al. 2020b; Ozturk and Silah 2020; Tolkou et al. 2023), pH 2.0 also found to be optimum for the removal of RBBR dye, as shown in Fig. 8. The results for all adsorbents used indicate that the adsorption efficiency decreased as solution pH increased, and at pH 2.0, banana peels had the highest removal rate at 100%, orange peels at 91%, and pomegranate peels at 70%. Under acidic conditions, strong electrostatic interactions occur between the cationic groups (ΝΗ_3_^+^) of adsorbents and the anionic groups of RBBR dyes. Under alkali conditions at pH 9.0, the adsorption efficiency significantly decreased to 58, 51, and 41% for banana, orange, and pomegranate peels, respectively, due to competitions between anionic RBBR dye ions and hydroxyl ions (Arya et al. 2020b; Yeow et al. 2021). Therefore, electrostatic attraction between the positively charged surface of banana, orange, and pomegranate peels at pH 2.0 and negatively charged RR120, RB5, and RBBR reactive dye molecules may be favored as the principal adsorption mechanism. Nevertheless, in Fig. 8, mainly in the case of banana and orange peels, a slight increase in the removal of anionic dyes is most likely observed above the pH_pzc_ value of each material. This fact is possibly attributable to the presence of some positively charged dye groups (ΝΗ_2_^+^) that interact with the negatively charged surface of adsorbents.

On the other hand, MB is a cationic dye (Fig. 1b), and it was also chosen in this study because of its strong adsorption on solid adsorbents as found in the literature (Atay 2018). MB occurs in the singly protonated form (MBH^2+^) in water due to the protonation of the dimethylamino groups (Minamoto et al. 2021). As derived from the pH drift method, the pH_pzc_ of banana peels was 6.06, of orange peels 4.09, and of pomegranate peels 3.33. As illustrated in Fig. 8, with the increase of pH value, an increase in MB dye removal was observed, for instance from 65 and 43% at pH 2.0, to 98% and 94% at pH 9.0 by using banana and orange peels, respectively. This could be due to the fact that the pH 9.0 of the solution was greater than the pH_pzc_ of the adsorbents, so the surface charge was negative, while in an acidic conditions, the positively charged surface of the adsorbent seemed to limit the adsorption of the cationic dye (Khuluk et al. 2019). Hence, an increase in electrostatic attraction between positively charged dye and negatively charged adsorbent obtained at pH 9.0. However, when the pomegranate peels were used as the adsorbent, it was observed that the removal rates of MB were high, at all pH values, with percentages ranging from 93% at pH 2.0 to 97% at pH 9.0. This is most likely due to the low pH_pzc_ displayed by pomegranate peels (3.33), and its surface charge changes to anionic at sufficiently acidic values, and therefore, from this value and above, electrostatic attractions are observed, between the anionic adsorbent and cationic MB dye ions.

Taking into account all the above, Table 3 summarizes the optimal conditions obtained during the study of the adsorption of the four dyes on the surface of the three natural adsorbents examined in this study. These conditions will be used in the following process evaluation experiments.

**Table 3:** Optimum selected pH values and dosage for further experiments

Dye	Banana		Orange		Pomegranate	
	pH	Dosage (g/L)	pH	Dosage (g/L)	pH	Dosage (g/L)
RR120	2.0	6.0	2.0	6.0	2.0	6.0
RB5	2.0	6.0	2.0	6.0	2.0	6.0
RBBR	2.0	5.0	2.0	6.0	2.0	6.0
MB	9.0	4.0	9.0	6.0	9.0	6.0

#### Effect of initial dye concentration

**Fig. 10:**
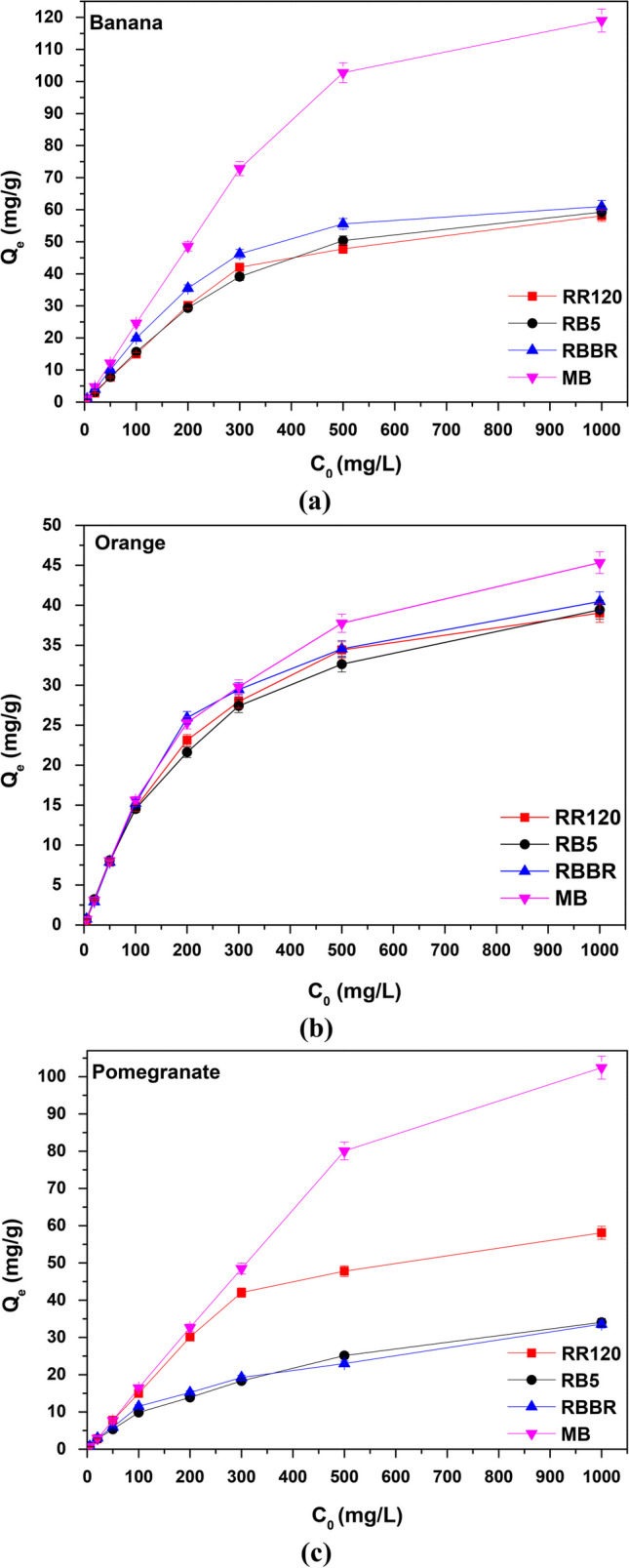
Effect of initial dye concentration onto **a** banana, **b** orange, and **c** pomegranate peel adsorption; *C*_0_ 5–1000 mg/L, *T* = 298 K, contact time 24 h, dose and pH according to Table 3 results

The effect of the initial concentration of RR120, RB5, RBBR, and MB on the adsorption efficiency of banana (Fig. 10a), orange (Fig. 10b), and pomegranate (Fig. 10c) peels was studied by changing the initial dyes’ concentration between 5 and 1000 mg/L and by appliyng the optimum selected pH values and dosages, as resulted in Table 3, for each dye solution and adsorbent. The data showed that there was a steady increase in adsorption capacity with the increase in dye concentration and that could be attributed to the fact that the driving force provided by the increased solute concentration which is sufficient to overcome the mass transfer resistance between the solid and liquid phases (Inglezakis et al. 2020). Therefore, adsorption will be enhanced with higher initial concentration (Banerjee and Chattopadhyaya 2017). The results were found to be in full agreement with the literature (Arya et al. 2020a; Moussavi and Mahmoudi 2009).

In particular, the adsorption capacity of banana peels (Fig. 10a) was found to increase remarkably from 1.1 to 119 mg/g for MB (pH 9.0 and 4.0 g/L) and from 0.8 to 60 mg/g for RR120 (pH 2.0 and 6.0 g/L), RB5 (pH 2.0 and 6.0 g/L), and RBBR (pH 2.0 and 5.0 g/L). In the case of orange peels (Fig. 10b), the adsorption capacity for all the dye solutions showed a lower increase ranging from 0.7 to 45 mg/g. Finally, the second most effective material after banana peels, the pomegranate peels, also showed an impressive increase in capacity with increasing initial concentration, especially of MB from 0.7 to 102 mg/g (pH 9.0 and 6.0 g/L), as shown in Fig. 10c. For RR120, the relative variations in adsorption capacity were from 0.5 to 58 mg/g (pH 2.0 and 6.0 g/L), for RB5 (pH 2.0 and 6.0 g/L) from 0.7 to 35 mg/g, and for RBBR (pH 2.0 and 6.0 g/L) from 0.8 to 34 mg/g, as initial solution concentration increases.

#### Effect of contact time

**Fig. 11:**
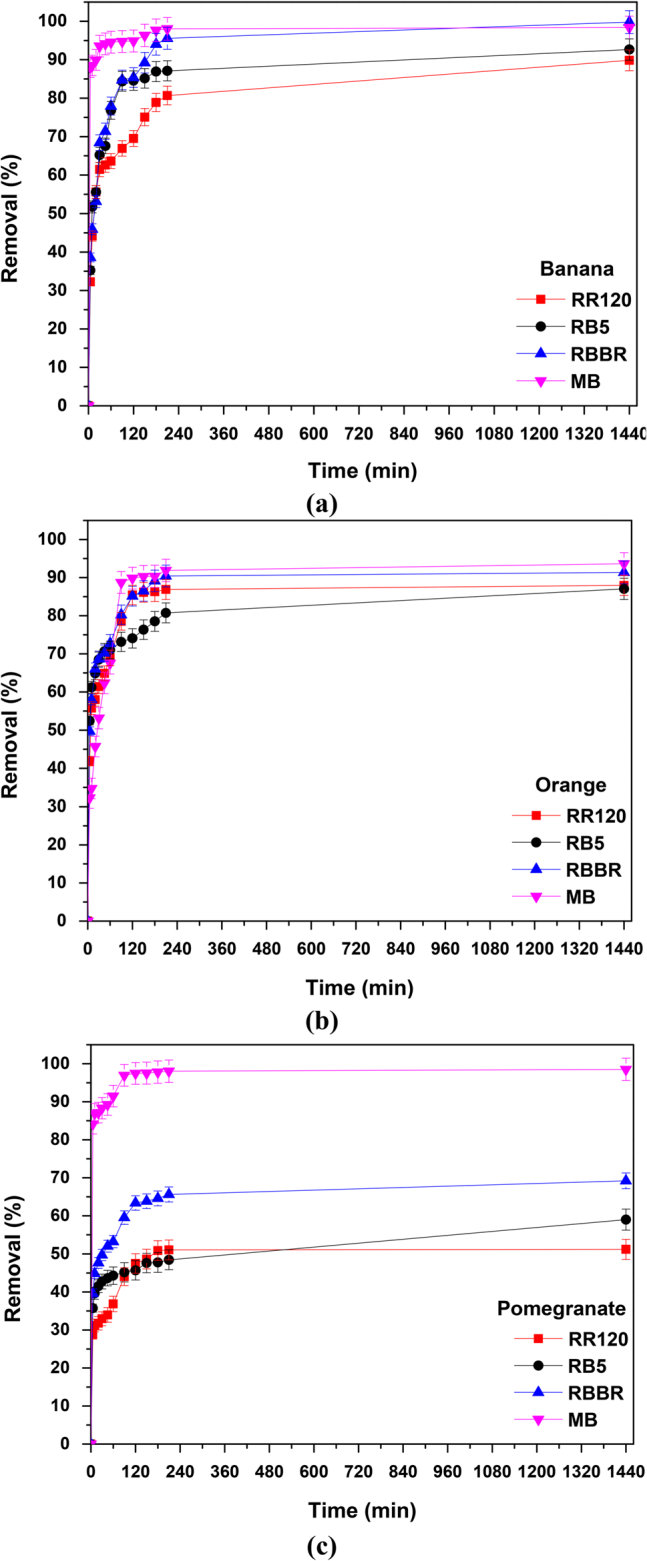
Effect of contact time on dye removal with **a** banana, **b** orange, and **c** pomegranate peels; *C*_0_ 100 mg/L, *T* = 298 K, contact time 24 h, dose and pH according to Table 3 results

In Fig. 11, the effect of contact time on the adsorption of RR120, RB5, RBBR, and MB on banana (Fig. 11a), orange (Fig. 11b), and pomegranate (Fig. 11c) peels is presented. The effect was examined in the range of 5 to 1440 min (24 h) and in almost all cases the 240 min (4 h) of reaction determined as the optimal contact time for further batch experiments (in which the equilibrium achieved), indicating a rapid distribution and surface adsorption.

Specifically, for MB removal, it was found that the adsorption was very quick when banana and pomegranate peels were used as the 90% of the dye was removed in the first 20–30 min, and after 24 h, the relative percentage was 99%. By using orange peels, 90 min is required for the 90% removal.

In addition, for the RB5 dye up to 90 min, there is an immediate adsorption of the dye on banana peels, as the 85% was removed (Fig. 11a). When using orange and pomegranate peels, the respective removal rates decrease and the required contact time increases (Fig. 11b, c). Furthermore, for RBBR, 85, 80, and 60% removal rates were achieved in the first 90 min for banana, orange, and pomegranate peels, respectively, reaching 100% with the use of banana after 360 min (6 h) up to 1440 min (24 h) where equilibrium occurred.

In the case of RR120 dye, more time (150 min) is required to remove the 75% of the color with banana peels and only 50% with pomegranate peels, with the latter being the highest percentage removed even after 24 h. Relative adsorption on orange peels was somewhat faster and more efficient in the first 90 min (80% removal), as shown in Fig. 11b.

### Adsorption isotherms

**Fig. 12:**
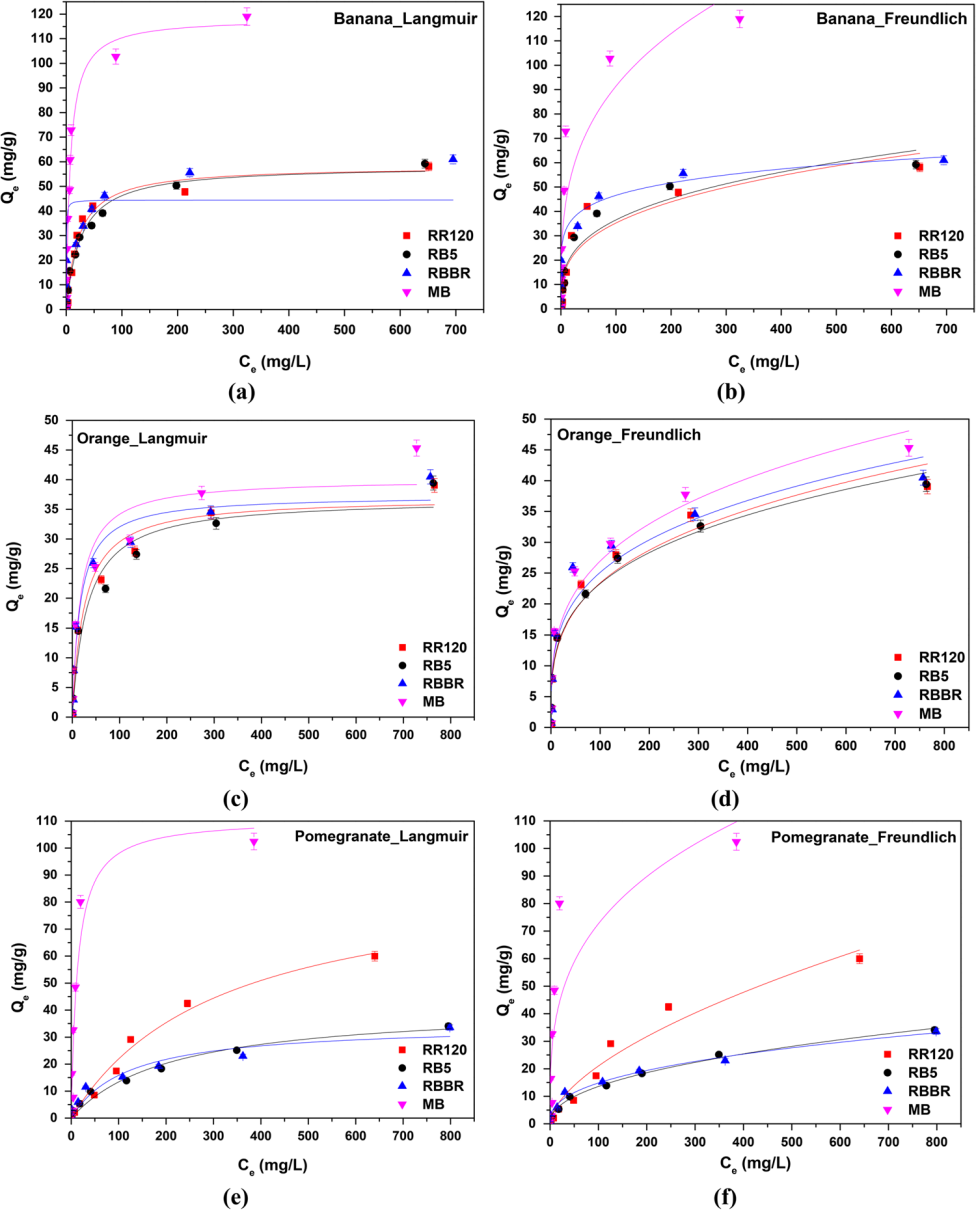
Langmuir and Freundlich isotherm models for the adsorption of dyes onto **a**, **b** banana, **c**, **d** orange, and **e**, **f** pomegranate peels. *C*_0_ 5–1000 mg/L, *T* = 298 K, contact time 24 h, dose and pH according to Table 3 results

Langmuir and Freundlich isotherm models were used in this study for the evaluation of the equilibrium adsorption data of RR120, RB5, RBBR, and MB onto banana, orange, and pomegranate peels at the optimum conditions. The obtained experimental data were fitted to those models, and the resulting non-linear plots are shown in Fig. 12, while Table 4 summarizes the relative equilibrium constants and coefficients. According to the results, the adsorption in some of the adsorbent materials and dyes fitted better the Freundlich and in some others the Langmuir isotherm model based on the values of the determination coefficient (*R*^2^). It should be noted here that the Freundlich adsorption isotherm model is valid for heterogeneous surfaces and large value of *n* indicates a strong interaction between the surface of the adsorbent and dyes (Arya et al. 2020a). On the other hand, Langmuir model suggests that monolayer adsorption of the dye on the homogeneous surface of the adsorbent takes place (Moussavi and Mahmoudi 2009).

**Table 4:** Constants of Freundlich and Langmuir isotherm models. *C*_0_ 5–1000 mg/L, *T* = 298 K, dose and pH according to Table 3 results. *C*_0_ 5–1000 mg/L, *T* = 298 K, dose and pH according to Table 3 results

Peels	Dye	Freundlich isotherm model				Langmuir isotherm model		
		1/*n*	*n*	*K*_*F*_ (mg/g)∙(L/mg)^1*/n*^	*R*^2^	*Q*_*m*_ (mg/g)	*K*_*L*_ (L/mg)	*R*^2^
Banana	RR120	0.3169	3.1559	8.1909	0.8265	58.24	0.0450	**0.9644**
	RB5	0.3099	3.2270	8.7671	0.9202	58.41	0.0379	**0.9835**
	RBBR	0.2042	4.8983	23.328	**0.9065**	44.47	0.0433	0.7911
	MB	0.3056	3.2721	22.373	0.8299	112.35	0.0912	**0.9673**
Orange	RR120	0.2959	3.3792	5.9862	0.9247	36.95	0.0040	**0.9554**
	RB5	0.2819	3.5470	6.3575	**0.9711**	36.79	0.0320	0.9175
	RBBR	0.2755	3.6293	7.0579	0.9048	37.32	0.0606	**0.9659**
	MB	0.2919	3.4260	7.0233	**0.9399**	40.13	0.0572	0.9221
Pomegranate	RR120	0.5928	1.6868	1.3692	0.9529	91.29	0.0032	**0.9836**
	RB5	0.5936	1.6846	1.6846	**0.9948**	41.74	0.0047	0.9711
	RBBR	0.3857	2.5930	2.5119	**0.9876**	34.72	0.0084	0.9229
	MB	0.3053	3.2754	17.8092	0.6903	98.08	0.0747	**0.8961**

In more details, regarding RR120 dye solution, it was found that the adsorption on all three examined adsorbents fitted better to Langmuir model, displaying a higher *R*^2^ (0.9644, 0.9554, and 0.9836 for banana, orange, and pomegranate peels, respectively), exhibiting a maximum adsorbent capacity, on the homogeneous surface of the adsorbent, of 58.24 mg banana/g, 36.95 mg orange/g, and 91.29 mg pomegranate/g. The latter is also in agreement with the literature for RR120 dye and its better correlation with Langmuir model (Navaei et al. 2019).

On the other hand, in the case of RB5, the results showed a better correlation to Freundlich model when orange (*R*^2^ = 0.9711) and pomegranate (*R*^2^ = 0.9948) are used and to Langmuir model in the case of banana peels (*R*^2^ = 0.9835). The RBBR dye, which belongs to the same category as RB5, followed the Langmuir model on orange peels with a higher *R*^2^ = 0.9659, but the Freundlich model on banana and pomegranate peels (*R*^2^ = 0.9065 and 0.9876, respectively). That could be attributed to the groups NH_3_^+^ existing on the dye (Fig. 1d), and have a strong affinity with the cationic exchange sites of banana peels (Arya et al. 2020a).

Finally, for MB dye solution, Freundlich model was better fitted in the case of orange peels used as adsorbent (*R*^2^ = 0.9399). Langmuir model was dominant in the case of banana (*R*^2^ = 0.9673) and pomegranate (*R*^2^ = 0.8961) peels, relative to literature (Hurairah et al. 2020), indicating a very high adsorption capacity, i.e., 112.35 mg banana/g and 98.08 mg pomegranate/g.

Consequently, it could be assumed that monolayer adsorption of the dye on the homogeneous surface of the adsorbent (Langmuir model) took place in the cases of RR120 on banana, orange, and pomegranate peels, of RB5 on banana peels, of RBBR on orange peels, and of MB on banana and pomegranate peels. On the contrary, multilayer adsorption with strong interactions between the heterogeneous surface of the adsorbent and dyes (Freundlich model) occurred in the cases of RB5 on orange and pomegranate peels, of RBBR on banana and pomegranate peels, and of MB on orange peels.

### Adsorption kinetics

**Fig. 13:**
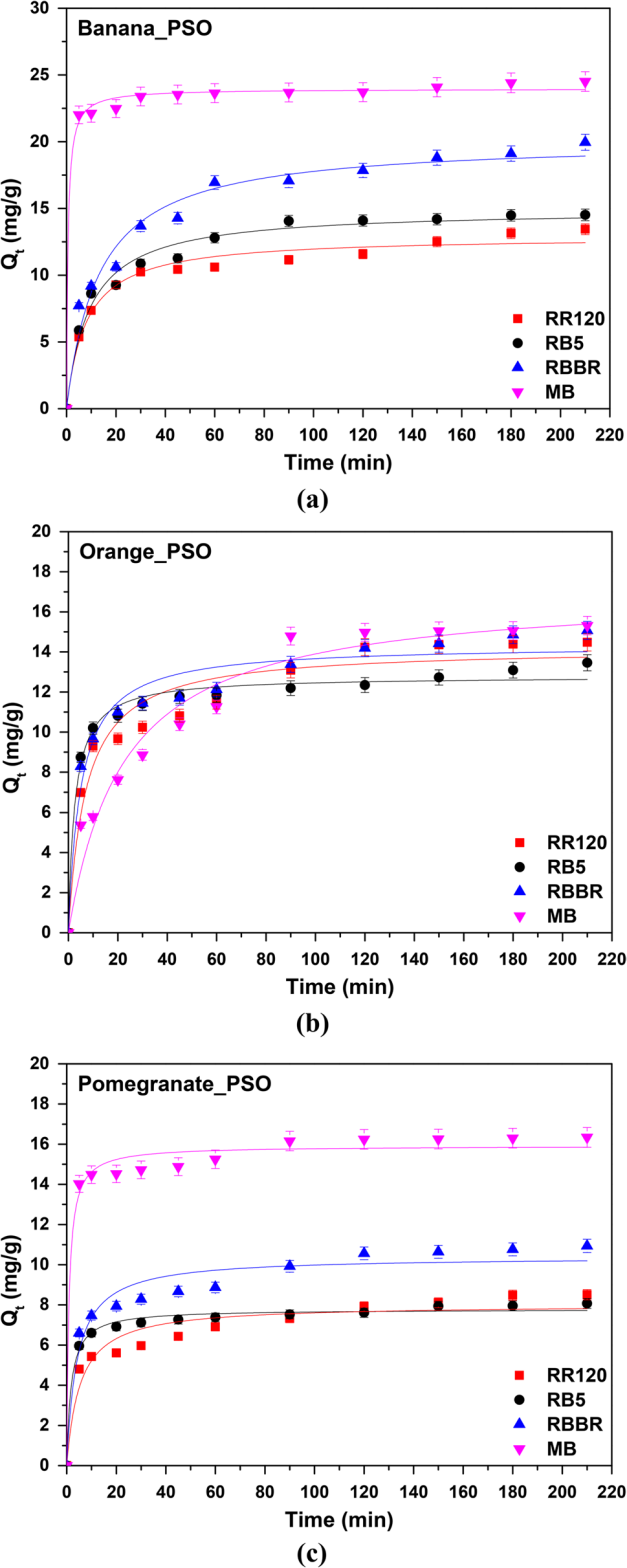
Kinetic pseudo-second-order models for the adsorption of dyes onto **a** banana, **b** orange, and **c** pomegranate peels adsorption; *C*_0_ 100 mg/L, *T* = 298 K, dose and pH according to Table 3 results

Adsorption kinetics is used to understand the rate of adsorption and the mechanism that controls the whole process. In order to interpret the kinetics of the adsorption of RR120, RB5, RBBR, and MB on banana (Fig. 13a), orange (Fig. 13b), and pomegranate (Fig. 13c) peel adsorbents, the pseudo-first-order (PFO) and the pseudo-second-order (PSO) kinetic models were used. According to the results, the adsorption of all the dyes examined on the surface of all the tested adsorbents was found to better fit to pseudo-second-order model, by comparing the *R*^2^ values (> 0.93) presented in Table 5. It should be clarified that the corresponding *R*^2^ values obtained during the application of the pseudo-first-order model were < 0.9, and for this reason, the relevant parameters are not shown in this paper. PSO model and its non-linear forms are described in Fig. 13 and Table 5. Moreover, the kinetic parameters showed that almost all *Q*_*e*,cal_ values calculated from Eq. 6 were comparable to the experimental values obtained from the adsorption studies of RR120, RB5, RBBR, and MB on banana, orange, and pomegranate peels. For instance in the case of MB dye, the *R*^2^ was the highest (0.9957) when banana peels were used as the adsorbent, and the relative *Q*_*e*,cal_ and *Q*_*e*,exp_ (calculated from Eq. 2) were 23.96 and 24.6 mg/g. Therefore, the adsorption kinetic study showed that the adsorption was closer most likely to chemisorption (Arya et al. 2020a; Tsoutsa et al. 2023).

**Table 5:** Pseudo-second-order kinetic parameters. *C*_0_ 100 mg/L, *T* = 298 K, dose and pH according to Table 3 results

Peels	Dye	*Q*_*e*,exp_ (mg/g)	Pseudo-second-order model		
			*K*_2_ (L/mg∙min)	*Q*_*e,*cal_ (mg/g)	*R*^2^
Banana	RR120	14.96	0.0972	12.91	0.9755
	RB5	15.44	0.0072	14.93	0.9788
	RBBR	19.96	0.0038	20.11	0.9693
	MB	24.60	0.0719	23.96	0.9957
Orange	RR120	14.66	0.0099	14.20	0.9403
	RB5	14.51	0.0298	13.78	0.9864
	RBBR	15.23	0.0140	14.35	0.9573
	MB	15.60	0.0026	16.04	0.9581
Pomegranate	RR120	8.53	0.0234	8.01	0.9358
	RB5	9.83	0.0729	8.78	0.9898
	RBBR	11.54	0.0233	10.39	0.9452
	MB	16.42	0.0708	15.91	0.9851

### Thermodynamics

**Fig. 14:**
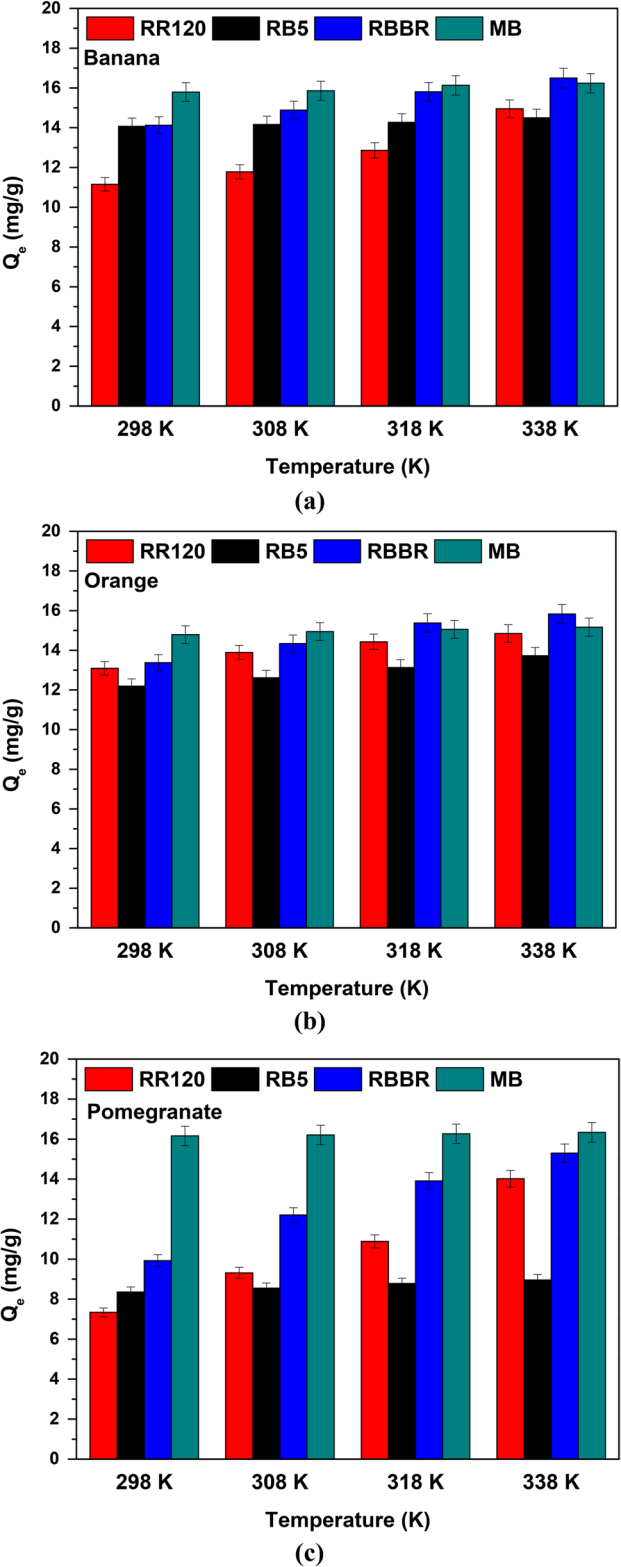
Effect of temperature on dye removal with **a** banana, **b** orange, and **c** pomegranate peels; *C*_0_ 100 mg/L, contact time 90 min, dose and pH according to Table 3 results

Figure 14 depicts the variation of *Q*_*e*_ (mg/g) with temperature in thermodynamic experiments. It is evident that as the temperature increases, the adsorption capacity also tends to increase. The improved adsorption and uptake efficiency of the dyes on the particle surface at higher temperatures indicate an endothermic nature of the adsorption process. The increase in *Q*_*e*_ at higher temperatures could be attributed either to the chemical interaction between the dye molecules and the adsorbents or to the formation of new adsorption sites on the surface of the particles (Parimelazhagan et al. 2022).

According to thermodynamics for banana peels, the *∆H°* and *∆S°* values were derived by examining the ln(*K*_*c*_) versus 1/*T* plot (Lima et al. 2019), exhibiting high correlation coefficients of *R*^2^ = 0.953, 0.991, 0.974, and 0.927 for RR120, RB5, RBBR, and MB, respectively. As shown in Table 6, the adsorption process for each dye resulted in negative *∆G°* values. This suggests that the removal of dyes through adsorption took place spontaneously and without the need for additional energy from an external source. The adsorption process of banana peels in RR120, RB5, RBBR, and MB was found to be endothermic due to the values of *∆H°* (31.299 kJ/mol, 4.471 kJ/mol, 61.539 kJ/mol, and 16.497 kJ/mol, respectively). The positive *∆S°* values observed for RR120 (0.1097 kJ/mol∙K), RB5 (0.0290 kJ/mol∙K), RBBR (0.2190 kJ/mol∙K), and MB (0.0792 kJ/mol∙K) are results of the enhanced disorder or randomness of the adsorbates at the interface between the solids and the solutes during the adsorption processes (Rao 2017).

**Table 6:** Thermodynamic parameters for the adsorption *C*_0_ 100 mg/L, contact time 90 min, dose and pH according to Table 3 results

Peels	Dye	*T* (K)	∆*G°* (kJ/mol)	∆*H°* (kJ/mol)	∆*S°* (kJ/mol∙K)	*R*^2^
Banana	RR120	298	− 1.405	31.299	0.1097	0.953
		308	− 2.503			
		318	− 3.600			
		338	− 5.795			
	RB5	298	− 4.159	4.471	0.0290	0.991
		308	− 4.448			
		318	− 4.738			
		338	− 5.317			
	RBBR	298	− 3.726	61.539	0.2190	0.974
		308	− 5.916			
		318	− 8.106			
		338	− 12.486			
	MB	298	− 7.108	16.497	0.0792	0.927
		308	− 7.900			
		318	− 8.642			
		338	− 10.276			
Orange	RR120	298	− 3.373	16.510	0.0667	0.958
		308	− 4.040			
		318	− 4.707			
		338	− 6.042			
	RB5	298	− 2.480	11.443	0.0467	0.996
		308	− 2.948			
		318	− 3.415			
		338	− 4.349			
	RBBR	298	− 3.586	33.052	0.1229	0.964
		308	− 4.816			
		318	− 6.045			
		338	− 8.504			
	MB	298	− 5.157	5.215	0.0348	0.966
		308	− 5.505			
		318	− 5.853			
		338	− 6.544			
Pomegranate	RR120	298	− 0.607	39.807	0.1312	0.993
		308	− 0.706			
		318	− 1.919			
		338	− 4.543			
	RB5	298	− 0.031	3.066	0.0104	0.965
		308	− 0.135			
		318	− 0.239			
		338	− 0.447			
	RBBR	298	− 1.089	42.479	0.1462	0.993
		308	− 2.551			
		318	− 4.013			
		338	− 6.937			
	MB	298	− 8.553	9.345	0.0601	0.996
		308	− 9.154			
		318	− 9.754			
		338	− 10.955			

For the orange peels, the relative plot showed strong correlation coefficients of *R*^2^ = 0.958, 0.996, 0.964, and 0.966 for RR120, RB5, RBBR, and MB, respectively. As presented in Table 6, the adsorption process for each dye exhibited negative *∆G°* values, indicating that the removal of dyes through adsorption occurred spontaneously without requiring additional external energy. The adsorption process of orange peels in RR120, RB5, RBBR, and MB was determined to be endothermic based on the respective *∆H°* values of 16.510 kJ/mol, 11.443 kJ/mol, 33.052 kJ/mol,, and 5.215 kJ/mol. The positive *∆S°* values observed were for RR120 0.667 kJ/mol∙K, for RB5 0.0467 kJ/mol∙K, for RBBR 0.1229 kJ/mol∙K, and for MB 0.0348 kJ/mol∙K, and were attributed to the increased disorder or randomness of the adsorbates at the interface between the solid and solute during the adsorption process. Due to the adsorbed species, the adsorbed solvent molecules are displaced and thus gain more translational entropy than is lost from the adsorbed ions/molecules, thus enhancing the appearance of randomness in the system (Saha and Chowdhury 2011).

Additionally, based on the identical graph, the values of *∆H°* (39.807 kJ/mol for RR120, 3.066 kJ/mol for RB5, 42.479 kJ/mol for RBBR, and 9.345 kJ/mol for MB) and *∆S°* (0.1312 kJ/mol∙K for RR120, 0.0104 kJ/mol∙K for RB5, 0.1462 kJ/mol∙K for RBBR, and 0.0601 kJ/mol∙K for MB) derived from the application of pomegranate peels were obtained, with an *R*^2^ of 0.993 for RR120, 0.965 for RB5, 0.993 for RBBR, and 0.996 for MB, as presented in Table 6. As it referred above, the positive entropy values indicate the heightened disorder or randomness of the adsorbates at the interface between solids and solutes during the adsorption processes, while the positive enthalpy values signify an endothermic reaction and the values of the free energy Gibbs (*∆G*^*0*^) were found to be negative showing that the adsorption took place spontaneously (Sun et al. 2021).

### Regeneration study

Regeneration studies were performed to investigate the reusability of banana, orange, and pomegranate peels in the removal of RB5 dye from wastewater. RB5 dye is one of the most widespread synthetic reactive dyes used in the dyeing industry as it is used to dye mainly several cellulosic fibers, such as cotton, wool, and nylon (El Bouraie and El Din 2016), and for this reason, it was chosen indicatively to study the regeneration and reusability of the peels. Therefore, each cycle was conducted under similar conditions, involving an initial RB5 concentration of 100 mg/L and an adsorbent dosage of 6.0 g/L at pH 2.0 ± 0.1 for each adsorbent for approximately 90 min. After the completion of the first cycle, the particles utilized in the process underwent a treatment using a 1 M NaOH solution. Following the treatment, the particles rinsed with distilled water to remove any remaining traces of the base. An appropriate duration was chosen so that it was possible to reuse the adsorbents in subsequent cycles, ensuring their effectiveness and maintaining their ability to remove RB5 from the wastewater.

**Fig. 15:**
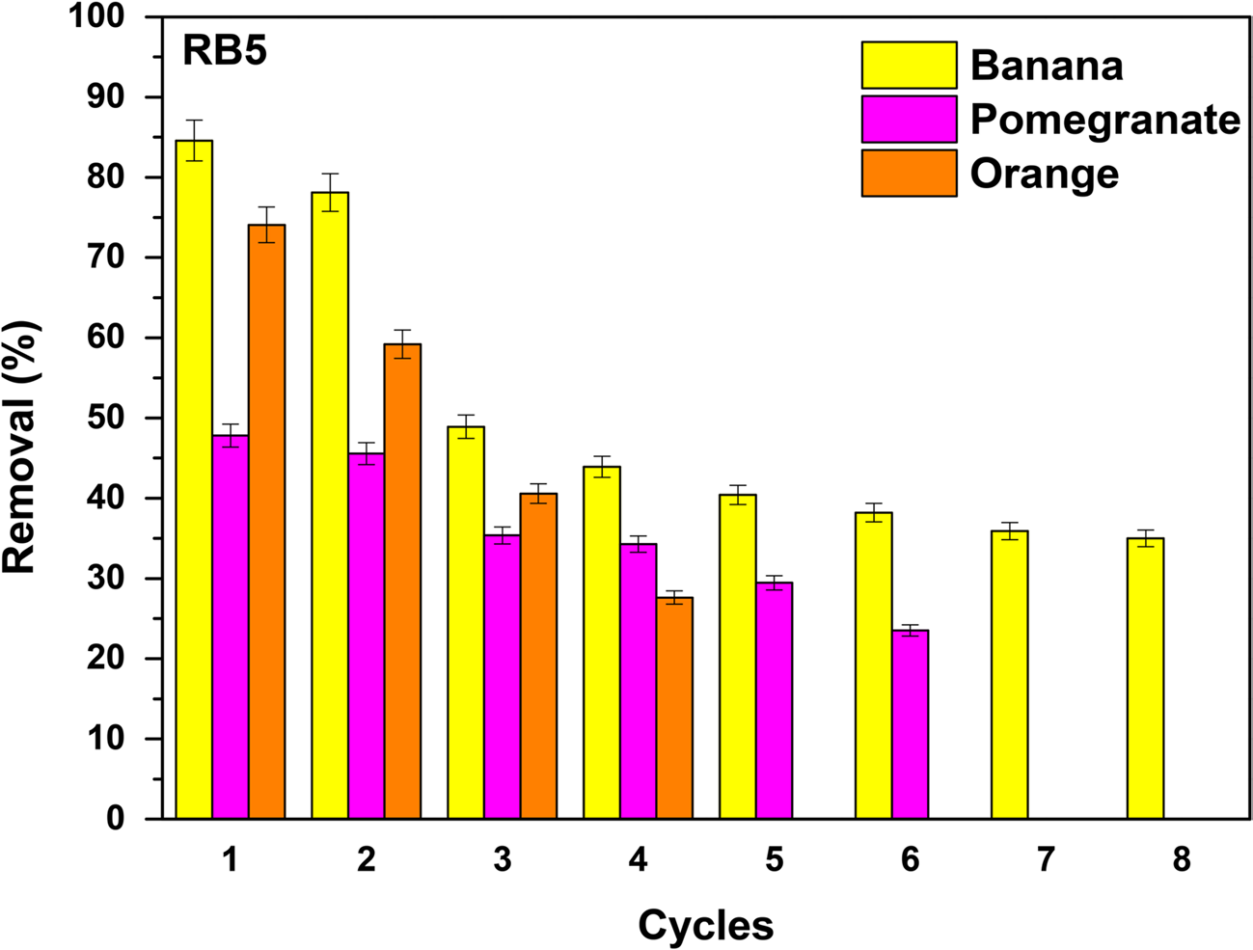
RB5 adsorption on banana, orange, and pomegranate peels; *C*_0_ 100 mg/L, pH 2.0 ± 0.1 dose 6.0 g/L, *T* = 298 K, contact time 90 min, for adsorption–desorption cycles after regeneration at alkalic pH values, by using 1 M NaOH treatment

As shown in Fig. 15, the regenerated banana peels could be effectively reused for 8 cycles in removing RB5, following treatment with 1 M NaOH. The percentage of RB5 removal was approximately 85% during the initial (first) cycle, after 90 min of treatment, gradually declining to around 35% by the eighth cycle. The orange peels found to be effective as adsorbent for RB5 removal after 4 cycles of reusability. During the first cycle, 74% of RB5 was removed, but this percentage gradually decreased to around 28% by the fourth cycle. The pomegranate peels regenerated up to 5 cycles with removal percentage of RB5 dye in the first cycle being 48% and in the fifth cycle 24%. In summary, it is clear the viability of reusing the banana, orange, and pomegranate peel adsorbents for 8, 4, and 5 cycles in the effective removal of RB5 from wastewater, showing a reduction of around 50% loss of their effectiveness.

### Mixtures of dyeing solutions

**Fig. 16:**
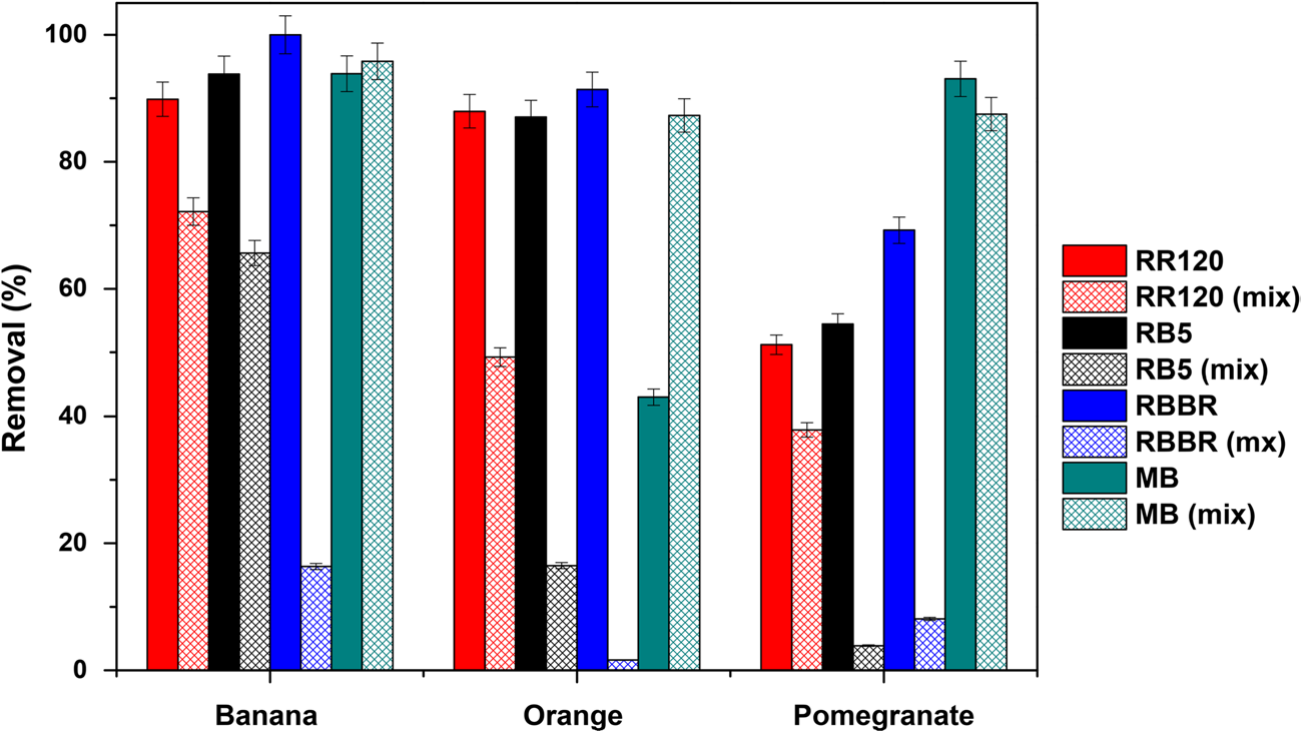
Comparison of natural adsorbents on RR120, RB5, RBBR, and MB removal, individual, and on mix dye solution. *C*_0_ 100 mg/L, pH 2.0 ± 0.1, dose 6.0 g/L, *T* = 298 K, contact time 24 h

The ability of banana, orange, and pomegranate peels to simultaneously remove basic cationic dyes (MB) and anionic dyes (RR120, RB5, and RBBR) in a mix solution containing 100 mg/L of each dye was investigated. The conditions applied were pH 2.0 ± 0.1, dose 6.0 g/L, and *T* = 298 K. The comparative results of natural adsorbents on dye removal are presented in Fig. 16, separately and together in solution. MB occurs in the single protonated form (MBH^2+^) in water (Minamoto et al. 2021), and the anionic dyes contain the sulfonic groups (dye-SO_3_^−^) (Munagapati et al. 2019). In addition, as derived from the pH drift method, the pH_pzc_ of banana peels was 6.06, of orange peels 4.09, and of pomegranate peels 3.33. Thus, at pH 2.0, the surfaces of the materials are positively charged.

As shown in Fig. 16, MB adsorption on banana and orange peels increased (from 94 to 96 and 43 to 87%, respectively) in the presence of anionic dyes (synergistic effect), but a competitive effect was determined, mainly between RB5 and RBBR with MB, resulting in a decrease in their respective percentages (from 94 to 65% for RB5 on banana and from 87 to 17% on orange, and for RBBR from 100 to 16% and from 91 to 2%, respectively), a phenomenon found also in the literature (Shirazi et al. 2019). In the case of pomegranate peels, the removal percentage of MB did not differ much in the presence of the negative dyes, but the competitive (antagonism) effect was intense for RB5 and RBBR. The RR120 dye exhibited similar results in mix solution.

### Comparison with recent literature

The adsorption efficiency of the fruit peels used in this study for the removal of several dye solutions is compared with some related materials published in the recent literature. Various parameters used for this comparison and the findings are presented in Table 7. As depicted, in the case of RR120 dye, in addition to banana, orange, and pomegranate peels, other peels have been also used, such as those of cactus or modified orange peels, e.g., with quaternary amine. In all cases, acidic conditions proved to be more effective (pH 2 or 3), as well as the adsorbent dose used was 6.0 g/L. Only in the case that the peels were modified, the dose was lower (2.0 g/L) and the adsorption capacity was high. Variation existed mainly in the initial concentration of the dye (Munagapati et al. 2019).

**Table 7:** Comparison of adsorption capacity and experimental conditions of the proposed peels as adsorbents in this study, with other relative adsorbents in literature, for dye removal

Dye	Adsorbents	*C*_0_ (mg/L)	Dosage (g/L)	pH_init_	*Q*_max_ (mg/g)	Removal (%)	Ref
RR120	Banana peel	300	6.0	3.0	26.9	-	Munagapati et al. (2018)
	Quaternary amine modified orange peel	300	2.0	2.0	344.8	91.0	Munagapati et al. (2019)
	Cactus Peel	40	6.0	3.0	2.1	99.4	Gebrezgiher and Kiflie (2020)
	Banana peel	100	6.0	2.0	58.2	89.8	Present study
	Orange peel	100	6.0	2.0	37.0	87.9	Present study
	Pomegranate peel	100	6.0	2.0	91.3	51.2	Present study
RB5	Potato peel	50	20.0	3.0	46.5	95.1	Samarghandy et al. (2011)
	Banana peel	100	6.0	2.0	58.4	93.8	Present study
	Orange peel	100	6.0	2.0	36.8	87.1	Present study
	Pomegranate peel	100	6.0	2.0	41.7	54.4	Present study
RBBR	Pineapple leaf	500	10.0	No adjustment	9.6	96.2	Rahmat et al. (2016)
	Lime peel	500	10.0	No adjustment	9.6	95.9	Rahmat et al. (2016)
	Ambarella peel	50	10.0	7.0	7.1	58.6	Chong and Hadibarata (2021)
	Banana peel	100	5.0	2.0	44.5	99.8	Present study
	Orange peel	100	6.0	2.0	37.3	91.4	Present study
	Pomegranate peel	100	6.0	2.0	34.7	69.2	Present study
MB	Mango peel	50	5.0	No impact	10.0	99.7	Phawachalotorn and Suwanpayak (2021)
	Pomegranate peel	50	5.0	11.0	102.3	99.8	Daud et al. (2019)
	Banana peel	50	20.0	No impact	23.91	94.8	Dey et al. (2017)
	Banana peel	100	4.0	9.0	112.4	98.4	Present study
	Orange peel	100	6.0	9.0	40.1	93.6	Present study
	Pomegranate peel	100	6.0	No impact	98.1	97.5	Present study

In the case of RB5 removal, not many peel applications were found in the literature. Similar results to the present research, regarding the adsorption capacity and rate, were also found using potato peels, but in this case, a much higher dose of adsorbent (20 g/L) and a lower initial dye concentration (50 g/L) were used (Samarghandy et al. 2011).

In contrast, more variety in the use of peels as adsorbents was found in the literature for RBBR removal, for example, pineapple leaf, lime peel, and ambarella peel. What emerged from their comparison with the materials of this research is that they showed a lower adsorption capacity, only 7–9 mg/g compared to 35–45 mg/g when using the proposed materials, and in fact in the case of ambarella peel (Chong and Hadibarata 2021), a high dose was used and low initial concentration, offering only 58% removal.

Finally, for the removal of MB, banana and pomegranate peels were also used in the literature, along with the use of mango peels. The following are worth noting: first, the banana peels used in the present study proved to be very effective in yielding high adsorption capacity (112.4 mg/g) at basic pH values and furthermore a very low dose (4.0 g/L) was used, especially compared to other research found in the literature that also used banana peels (Dey et al. 2017). In that case, 20 mg/L was used with 50 mg/L initial dye concentration and only 23 mg/g uptake.

In conclusion, the materials used in this research (banana, orange, and pomegranate peels) proved to be effective in agreement with the literature. In addition, these are materials that promote the sustainability, economic use, and reuse of food waste, and in fact, as proposed in this study, by increasing the drying of the peels in air conditions, instead of extensive drying in an oven, the energy costs for the synthesis of the materials are reduced.

## Conclusions

In this study, banana, orange, and pomegranate peels were collected from local markets and prepared by a proposed simple and environmentally friendly method, promoting the sustainability, economic use, and reuse of food waste and applied as natural adsorbents for the removal of both anionic (RR120, RB5 and RBBR) and cationic (MB) dyes from wastewater. The application of such processed materials for the simultaneous removal of a wide variety of dyes is an innovative solution presented in this work. According to the characterizations from BET analysis, banana peels have the largest surface area and total pore volume (73.2 m^2^/g and 0.231 cm^3^/g, respectively).

Regarding the results, the optimum conditions found to be for banana peels: pH 2.0, 6.0 g/L for RR120 (90%) and RB5 (94%); 5.0 g/L for RBBR (100%); and pH 9.0, 4.0 g/L for MB (98%); orange peels: pH 2.0, 6.0 g/L for RR120, RB5, RBBR (88, 87, and 91%, respectively) and pH 9.0, 4.0 g/L for MB (94%); and pomegranate peels: pH 2.0, 6.0 g/L for RR120, RB5, RBBR (51, 59, and 69% respectively), and pH 9.0, 6.0 g/L for MB (98%). Moreover, the adsorption process in all cases was found to better fit to pseudo-second-order model, and according to isotherms, Freundlich model fitted better in some cases and the Langmuir model in others. Thermodynamics indicated that the adsorption was endothermic and that took place spontaneously, in all cases. According to regeneration experiments, this study demonstrates the viability of reusing the banana, orange, and pomegranate pee adsorbents for eight, four, and five cycles, showing a reduction of around 50% of their effectiveness. Moreover, the ability of banana, orange, and pomegranate peels to simultaneously remove cationic and anionic dyes in a mix solution was investigated. It was found that the MB adsorption on banana and orange peels increased in the presence of anionic dyes (synergistic effect).

In conclusion, the outcomes of the present study suggest that fruit peels can be used as sustainable natural adsorbents, for the removal both of cationic and anionic dyes from wastewater samples and that the simultaneous removal of dyes from mixtures should be further examined and analyzed. The fact that anionic dyes show a minimum of removal efficiency at specific pH values is a phenomenon that will concern our group at future studies.

## Data Availability

The datasets generated during and/or analyzed during the current study are available from the corresponding author on reasonable request.
